# The Evolution of Tyrosine-Recombinase Elements in Nematoda

**DOI:** 10.1371/journal.pone.0106630

**Published:** 2014-09-08

**Authors:** Amir Szitenberg, Georgios Koutsovoulos, Mark L. Blaxter, David H. Lunt

**Affiliations:** 1 Evolutionary Biology Group, School of Biological, Biomedical and Environmental Sciences, University of Hull, Hull, United Kingdom; 2 Institute of Evolutionary Biology, The University of Edinburgh, Edinburgh, United Kingdom; Tel Aviv University, Israel

## Abstract

Transposable elements can be categorised into DNA and RNA elements based on their mechanism of transposition. Tyrosine recombinase elements (YREs) are relatively rare and poorly understood, despite sharing characteristics with both DNA and RNA elements. Previously, the Nematoda have been reported to have a substantially different diversity of YREs compared to other animal phyla: the *Dirs1*-like YRE retrotransposon was encountered in most animal phyla but not in Nematoda, and a unique *Pat1*-like YRE retrotransposon has only been recorded from Nematoda. We explored the diversity of YREs in Nematoda by sampling broadly across the phylum and including 34 genomes representing the three classes within Nematoda. We developed a method to isolate and classify YREs based on both feature organization and phylogenetic relationships in an open and reproducible workflow. We also ensured that our phylogenetic approach to YRE classification identified truncated and degenerate elements, informatively increasing the number of elements sampled. We identified *Dirs1*-like elements (thought to be absent from Nematoda) in the nematode classes Enoplia and Dorylaimia indicating that nematode model species do not adequately represent the diversity of transposable elements in the phylum. Nematode *Pat1*-like elements were found to be a derived form of another *Pat1*-like element that is present more widely in animals. Several sequence features used widely for the classification of YREs were found to be homoplasious, highlighting the need for a phylogenetically-based classification scheme. Nematode model species do not represent the diversity of transposable elements in the phylum.

## Introduction

### Transposable elements

Transposable elements (TE) are mobile genetic elements capable of propagating within a genome and potentially transferring horizontally between organisms [1]. They typically constitute significant proportions of bilaterian genomes, comprising 45% of the human genome [2], 22% of the *Drosophila melanogaster* genome [3] and 12% of the *Caenorhabditis elegans* genome [4]. TEs may also have important evolutionary effects, such as promoting alternative splicing [5], inducing variation accumulation under stress [6] and increasing the genetic load [7].

TEs can be broadly divided into DNA and RNA classes. DNA TEs (transposons) transfer as dsDNA, leaving a vacant locus at the point of origin, together with a target site duplication (TSD) [7]. They are thought to increase in copy number via various recombination related mechanisms between vacant and populated TE loci, partly due to the similarity of TSDs across the genome [7]. RNA TEs (retrotransposons and retroposons) do not exit their locus of origin but rather propagate through the reverse transcription of an RNA intermediate copy back into an additional site in the genome [8]. RNA elements are usually the most numerous type of TE and can have tens of thousands, or even millions, of copies in a single genome [8]. Despite this there can be variation in the relative proportions and some species have DNA elements as the most frequent class, as the case is in *C. elegans* [9].

### Tyrosine recombinase TEs

Tyrosine Recombinase Elements (YREs) are found in both the DNA and RNA TE classes. They contain a Tyrosine Recombinase (YR) domain that replaces the transposase and integrase proteins encoded in DNA and RNA TEs, respectively. The YR domain facilitates transposition without forming a TSD. YREs have been suggested to have emerged from a single or several events of recombination between DNA and RNA elements [10] which makes them interesting and important group for understanding the evolution and maintenance of TEs more generally. YREs are diverse in sequence and structure, but this diversity is not equally represented across the animal phyla [10]–[12], and their evolutionary history can sometimes be puzzling. Nematoda for example are described as having one unique form of YREs (a form of *Pat1* found only in this phylum) and to entirely lack another (*Dirs1*), which is otherwise relatively common [12]. However, the diversity of YREs in Nematoda is still poorly understood and a phylogenetically informed analysis with broad taxonomic sampling of both the YREs and their hosts is required to thoroughly address the subject.

### YRE classification

DNA YREs possess only a YR protein domain and include the Crypton and TEC elements. Although Cryptons were first discovered in fungi [13], four distinct, possibly polyphyletic, lineages have been defined in fungi, diatoms and animals [10]. It is thought that Cryptons may have contributed to the origin of RNA YREs [10]. TEC elements, by contrast, appear to have a very limited taxonomic distribution and are currently known only from ciliates [14], [15].

**Figure 1:**
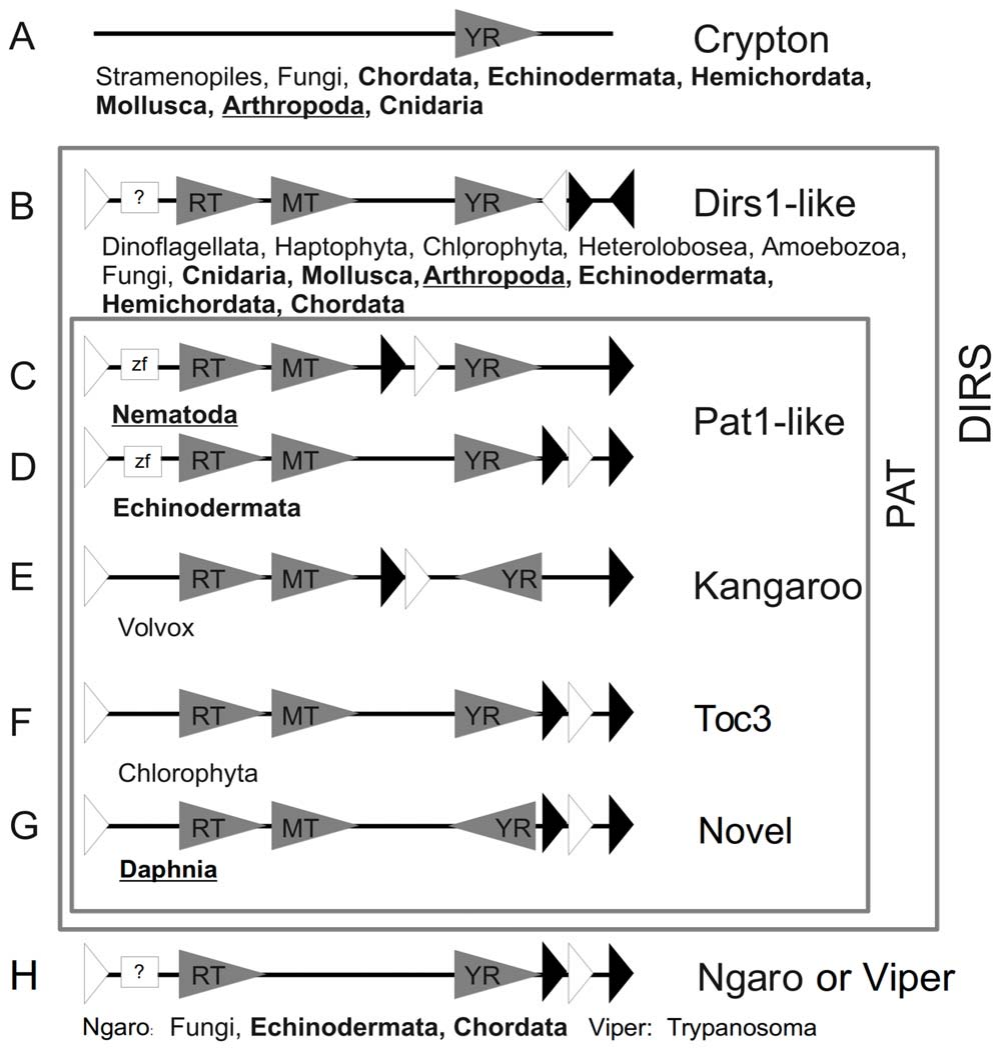
The diversity of tyrosine recombinase elements (YREs) and their diagnostic features for taxonomic classification. The known taxonomic distribution of each element (A–H) is listed along with a cartoon of its structure. Metazoa are in bold font and Ecdysozoa are underlined. The features considered are the presence and absence of the reverse transcriptase (RT), methyltransferase (MT) and tyrosine recombinase (YR) domains and their orientation (grey triangles), as well as the presence, absence and position of split direct repeats (pairs of triangles, sharing a colour and pointing in the same orientation), inverted repeats (pairs of triangles, sharing a colour and pointing in opposite orientation) and zinc finger motifs from the Gag protein. Where a question mark is indicated, some members of the group possess and others lack a zinc finger motif.

RNA YREs, like other long terminal repeat (LTR) retrotransposons, possess the capsid protein Gag, and a polyprotein that includes the reverse transcriptase (RT) and RNase H (RH) domains. LTR retrotransposons (*Gypsy*, *Copia* and BEL) may have been the source of the ancestral RNA element of the YRE ancestor [10]. Unlike the LTR retrotransposons, YRE retrotransposons possess the YR domain and lack the integrase gene [11], [16]. They sometimes also encode a methyltransferase (MT) domain (Figure 1).

### Structure based classification of YREs

A set of molecular sequence features are widely used to classify YRE retrotransposons: the presence and strand of the RT, MT and YR domains, the presence of a zinc finger (ZF) motif in the Gag protein, and the presence and relative arrangement of characteristic repeat sequences [12], [17]–[21] (Figure 1). Three groups of YRE retrotransposons have been defined: DIRS, Ngaro and Viper [19], [22].

DIRS elements are YRE retrotransposons that encode a putative MT domain. DIRS elements can be classified into *Dirs1*-like elements and PAT elements. PAT elements can be further classifieds into *Pat1*-like elements, *Toc* elements and *Kangaroo* elements (Figure 1). Within the DIRS group. *Dirs1*-like elements and PAT elements are differentiated by the presence of two consecutive pairs of inverted repeats in *Dirs1*-like (Figure 1B) and split direct repeats in PAT elements (Figure 1C–1F). *Dirs1*-like elements were discovered in Amoebozoa [17] and are also present in Viridiplantae, Metazoa and other eukaryotes. [12]. Like other YRE retrotransposons they have internal repeats that couple with the terminal ones (Figure 1B). For a detailed description of the repeat sequences of *Dirs1*, see Piednoël et al. [12].

**Figure 2:**
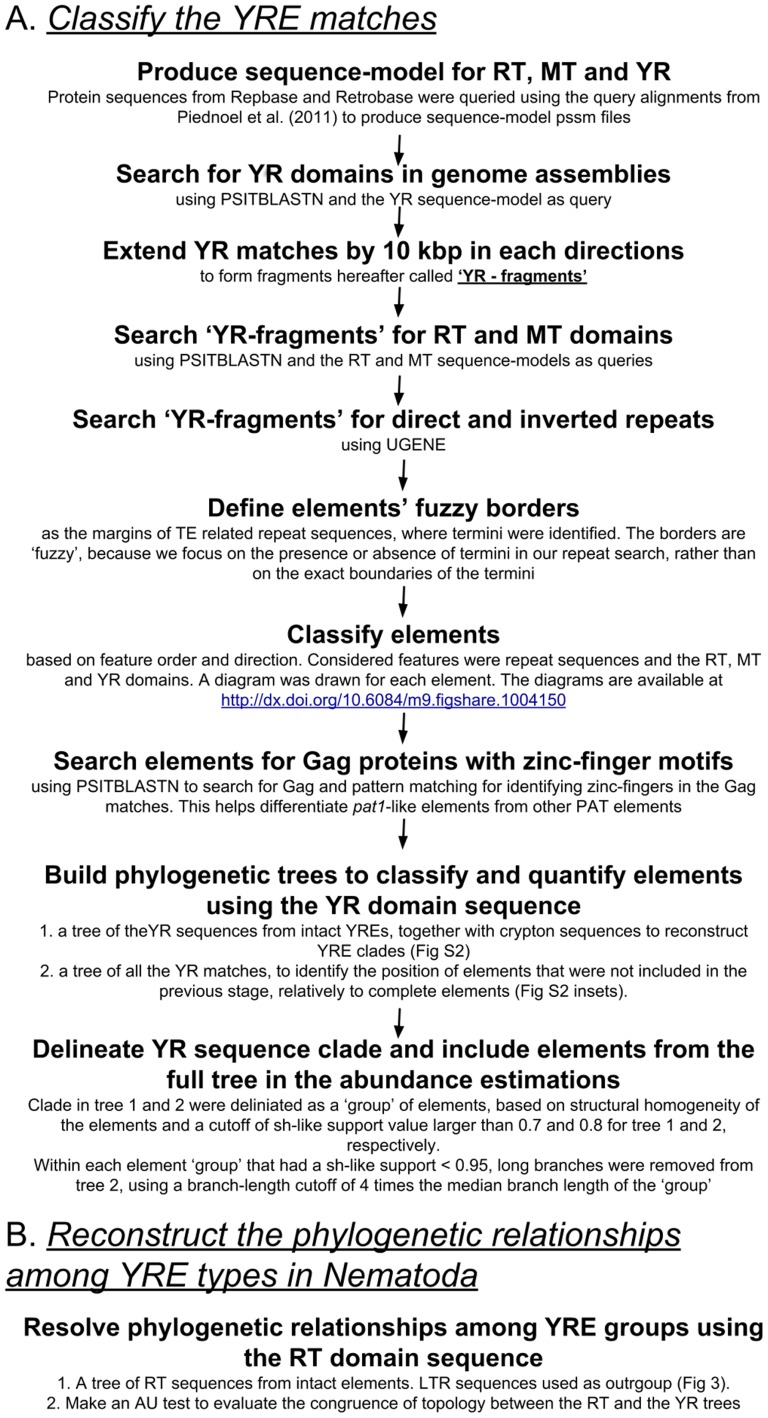
Schematic description of the workflow utilized in this study. A flow chart of the analysis steps described in the Material and Methods section, including the homology searches for YRE protein domains, the classification of YREs based on their features, the phylogenetic reconstruction of YRE relationships and their phylogenetic classification.

PAT elements are part of the DIRS group and include *Pat1, Toc* and *Kangaroo* elements (Figure 1C–1F). They differ from *Dirs1*-like elements by the presence of split direct repeats. These repeat sequences are also referred to as A1-B1-A2-B2 repeats where A2 is an identical repeat of A1 and B2 of B1. *Pat1* elements (Figure 1C–1D) were first identified in the nematode *Panagrellus redivivus* (Panagrolaimomorpha; Tylenchina; Rhabditida) [23] (see Figure 2 for relationships of species analysed) and subsequently also in the nematodes *Caenorhabditis briggsae* (Rhabditomorpha; Rhabditina; Rhabditida) [19] and *Pristionchus pacificus* (Diplogasteromorpha; Rhabditina; Rhabditida) [12]. A distinct form of *Pat1*-like elements was described from the sea urchin *Strongylocentrotus purpuratus* (Echinodermata) [19]. The nematode-form *Pat1*-like elements (Figure 1C) differ from the echinoderm-form (Figure 1D) in the placement of their internal repeat (A2B1) sequences. Both forms have a zincfinger motif in the Gag protein, which is absent from the other PAT elements, *Kangaroo* and *Toc*. The *Kanagaroo* element, found in *Volvox carteri* (Chlorophyta; Viridiplantae) [24], differs from other PAT elements by having an inverted YR domain (Figure 1E) and by the absence of a zinc finger motif. In *Kangaroo* elements, the internal repeats are located between the MT and YR domains (as observed in the nematode-form *Pat1*-like elements). *Toc3* PAT elements (Figure 1F) were found in algae [19] and differ from *Pat1*-like elements by the absence of a zinc-finger motif, and from *Kangaroo* elements by the direction of the YR domain.

Ngaro and Viper are two groups of non-DIRS YRE retrotransposons. These predominantly differ from DIRS elements by the absence of the putative MT domain (Figure 1H). Like PAT elements, from within the DIRS group, they possess split direct repeats, with the internal repeats found downstream to the YR domain [16], [19]. Ngaro elements were originally found in *Danio rerio* (zebrafish; Osteichthyes; Chordata), *S. purpuratus* and fungi [11], while Viper elements are found in *Trypanosoma* (Trypanosomatidae; Kinetoplastida) [22].

In spite of their exceptional diversity, YREs are rare compared to non-YRE transposable elements. They have been identified in few species, and, when present, they are found in low numbers: *Dirs1* from *Dictyostelium discoideum* (Amoebozoa [17]) is present in 40 intact copies and 200–300 fragments. Crypton (Figure 1A) is present in a few dozen copies in each of a range of eukaryote species [10]. TEs with such small population size, however, will be subject to strong genetic drift and variation in copy number, and thus will be prone to elimination [25]. Nematoda are believed to have undergone a shift in their YRE content compared to other phyla, losing *Dirs1*-like elements (Figure 1B) and accumulating *Pat1*-like elements [12]. However, the true diversity of YREs in Nematoda in not known as current estimates are based largely on a few, relatively closely related species (*P. redivivus*, *P. pacificus* and *C. elegans*). Here we survey whole genome sequencing data from a wide taxonomic range of nematode species and show that a shift in YRE content has indeed occurred. We found that *Dirs1*-like elements are present in at least one of the three Nematoda classes, and the nematode form of *Pat1*-like elements is closely related to *Pat1* elements from other animal phyla.

## Material and Methods

To identify and quantify YREs in nematodes, we utilized homology based search methods to locate YREs, made a preliminary classification based on characteristic features, and used phylogenetic methods to refine and corroborate these classifications. We conducted further phylogenetic analyses to classify partial or degenerate elements relative to complete elements. This stage allowed us to include partial and potentially degraded elements in the copy number counts and have a better understanding of the origins of the distribution of YREs among the nematode species. Unlike similarity based clustering methods (e.g., [12], [21], [26], [27]), a phylogenetic approach accounts for homoplasy and is better adapted for the analysis of potentially degraded sequences. The flow of our analysis is illustrated in Figure 2. In order to facilitate replication and extension of our work with new genomic data we have made all our analysis steps reproducible through use of an iPython notebook and github repository that include all the analysis code (http://dx.doi.org/10.6084/m9.figshare.1004150). A static html file of the notebook is included as Methods S1.

With the exception of genome assemblies, the repository includes all the input files. URLs to the genome assemblies are provided in Table S1. All the analyses and figures presented here can be reproduced by downloading the assembly files and executing the IPython notebook cells in sequence while following the instructions included in the notebook. However, since the assembly versions that were used here may be inaccessible in the future, all the pipeline's outputs are also provided in the github repository.

### Taxon sampling

Our nematode species sampling consisted of 34 genome assemblies belonging to ten orders and superorders. Most of the species (30) belong to the subclass Chromadoria, three to the subclass Dorylaimia and one to Enoplia. Five ecdysozoan species, including four arthropods and a single tardigrade, were selected as outgroup taxa. Non-ecdysozoan outgroup species included a cnidarian, two molluscs, an amoebozoan and three plants. The species and sources are listed in Table S1.

In addition to genome assemblies, we also analysed the Repbase Crypton and DIRS datasets [28], the Retrobase DIRS dataset (http://biocadmin.otago.ac.nz/fmi/xsl/retrobase/home.xsl), four *Pat1*-like elements from *P. pacificus* kindly provided by M. Piednoël, and the first *Pat1* sequence to have been described (Genbank accession X60774). These sources together formed our reference dataset. We examined the validity of element classifications produced by the pipeline using these known elements and also for seeding query alignments.

### Homology search based YRE identification

In order to find YREs in the assemblies we used a strategy modified from Piednoël et al. [12] (Figure 2A). First, we searched for YR domains in each whole genome assembly. YR matches were extended by 10 kb in each direction or to the contig end, whichever was encountered first. We then searched for RT and MT domains and direct and inverted repeats in the resulting sequences. This approach efficiently streamlined the homology searches while including only RT and MT domains that are likely to belong to YREs. The homology searches were conducted using PSITBLASTN [29], [30] with an expected value threshold of 0.01. The query models for these searches were seeded with the alignments from Piednoël et al. [12] and were extended by adding protein sequences from the reference dataset through PSIBLASTP search [29], [30].

Direct and inverted repeats on the extended YR fragments were detected with the BLAST based program UGENE [31], with only identical repeats at least 20 bp long allowed. These values represent the minimal repeat sequence in the results of Piednoël et al. [12]. Each annotated fragment was subsequently programmatically given a preliminary classification based on its similarity to the structures illustrated in Figure 1.

### Zinc finger motif pattern matching

Among PAT elements (Figure 1), only *Pat1* elements have zinc finger motifs in their Gag sequence [11]. Gag sequences from two *Pat1* elements were used to query the reference databases to produce a Gag sequence model using PSIBLASTP [29], [30] (Figure 2A). The sequences that were eventually used to produce the model represented all DIRS element diversity. PSITBLASTN [29], [30] was used to recover Gag sequences from the YRE DNA sequences found in the previous stage, with an expected value threshold of 0.01. The Gag sequences detected were searched for the zinc finger sequence patterns described by Poulter and Goodwin [11] using a python script (see Methods S1). The classification process was extended using a phylogenetic approach to include partial and degraded elements as well as complete ones.

### Phylogenetic reconstruction of YRE relationships

For the inference of phylogenetic relationships among YRE clades we considered only YRE matches that had at least YR and RT domains as well as terminal repeats. The RT domain may have had a different history from that of the YR domain as published YR and RT trees do not seem to be congruent [10], [32]. Therefore, a reciprocal AU-test for partition homogeneity was conducted in CONSEL 0.2 [33], using a RT, YR and combined datasets with identical YRE representation. Since the results indicated incongruence between the partitions (see Results and Methods S1), and since preliminary analysis revealed better sh-like support values in the tree that was reconstructed from the RT dataset, the RT domain was chosen for the phylogenetic reconstruction of YRE relationships (Figure 2B). *Gypsy*, *Copia* and BEL sequences from Repbase were added to the RT dataset prior to the analysis. The RT sequences were aligned with MAFFT 7 [34], [35] using default settings and then trimmed with TrimAl 1.2 [36] to remove positions with over 0.3 gap proportion. The tree was reconstructed using FastTree 2.1.7 [37] with gamma distribution of among site rate variation and with the JTT matrix of substitution rates. SH-like values were used as branch support, as they have been found to be highly correlated with bootstrap approaches and are rapidly calculated [38] (see Methods S1 for the exact command line parameters used).

### Phylogenetic approach to YRE classification and quantification

We chose a phylogenetic approach to element classification over genetic-distance clustering methods to better account for homoplasy in our sequence data (Figure 2A). Similar methods to the ones above were used to reconstruct two additional phylogenetic trees for the purpose of classification and quantification. The first tree was reconstructed from a dataset including only YR sequences from complete RNA YREs as well as Crypton YR sequences. This tree was used to delineate element clades. Only clades with sh-like support of 0.7 or above were considered, if they did not have conflicting YRE features based classifications. YR domain hits from reference elements helped to confirm the identity of the element clades.

The second tree included all the YR domain hits from both complete and truncated or degraded elements as well as YR sequences from Crypton elements (Figure 2A). This tree was used to identify the phylogenetic position of degraded and truncated elements relatively to complete elements and adjust their count accordingly, for each of the clades recovered in the previous tree. Only truncated or degraded elements that clustered with complete elements with sh-like support of 0.9 or above were considered. However, we have detached nodes with long branches from clades that included complete elements and had sh-like value <0.95, to avoid artifactual groupings. The branch-length cutoff that was used for node removal due to a long branch was four times the median branch-length of that clade.

### Assessment of the reliabilty of YRE counts

Given that the originating genome does in fact contain YRE elements, draft genome assemblies could be missing YRE elements for two reasons: The first is that by being incomplete they may stochastically miss some elements. The second reason arises from the assembly algorithms used, where highly similar elements may yield assembly graphs that the algorithm rejects as being too complex, or of too high coverage, to include in the reported assembly contigs. Since YREs often have a low copy number [10], [17] the second artefact is less likely, but a record of absence may simply reflect assembly quality. However, LTR retrotransposons are not likely to be absent from eukaryotic genomes and an inability to detect LTR elements would suggest that the assembly is simply not of sufficient quality. Therefore, in each of the species studied, we performed three additional PSITBLASTN [29], [30] searches for RT domains of *Gypsy*, *Copia* and BEL LTR retrotransposons. The query alignments were constructed in the same manner as described above and are available in the github repository.

## Results

We identified putative homologues of three YRE protein domains, YR, RT and MT, in genome assemblies of 34 nematode and 12 outgroup species. Over 2,500 significant matches to YREs were found in 24 species (Table 1). These were first classified based on the presence, absence and orientation of YRE sequence features (Figure 1). Although only 207 elements in 13 of the assemblies could be classified unequivocally based on these diagnostic features, these classified sequences were useful additional reference sequences, complementing the ones obtained from Retrobase (http://biocadmin.otago.ac.nz/fmi/xsl/retrobase/home.xsl) and Repbase-update [28] (Figure 2). In addition, we used them to corroborate the results of subsequent phylogenetic analyses.

**Table 1:** Mean contig lengths, contig length at N50 and PSIBLASTN putative transposable element counts of the genomes analysed.

Code	Species	Mean contig length	N50 contig length	YRE matches	Intact YREs	Partial YREs (Total)	BEL	Copia	Gypsy
Haor	*Howardula aoronymphium*	411	429	7	0	0	43	4	62
Ebre	*Enoplus brevis*	477	506	459	0	9(9)	513	50	535
Ovol	*Onchocerca volvulus*	1,146	1265	1	0	0	61	0	2
Mflo	*Meloidogyne floridensis*	1,231	3516	0	0	0	100	0	90
Cang	*Caenorhabditis angaria*	3,062	79858	11	0	2(2)	20	0	14
Wban	*Wuchereria bancrofti*	3,149	5161	0	0	0	65	0	3
Ooch	*Onchocerca ochengi*	3,970	12317	0	0	0	156	0	1
Otip	*Oscheius tipulae*	3,998	13984	12	0	0	10	1	8
Dsim	*Drosophila simulans*	4,029	7074	0	0	0	557	114	642
Hcon	*Haemonchus contortus*	4,991	13338	40	7	11(18)	582	0	448
Dimm	*Dirofilaria immitis*	5,498	71281	1	0	0	46	0	0
Rcul	*Romanomermis culicivorax*	5,580	20133	267	6	64(70)	538	9	682
Agam	*Anopheles gambiae*	7,667	1505544	1	0	0	664	263	654
Asuu	*Ascaris suum*	8,420	290558	1	0	0	62	0	47
Minc	*Meloidogyne incognita*	8,607	12786	0	0	0	157	1	91
C5sp	*Caenorhabditis* sp. 5	8,636	25228	0	0	0	98	0	50
Cjap	*Caenorhabditis japonica*	8,835	94149	62	2	48(50)	206	4	261
Tspi	*Trichinella spiralis*	9,256	6373445	0	0	0	220	29	140
Ppac	*Pristionchus pacificus*	9,539	1244534	57	7	26(33)	156	0	124
Dviv	*Dictyocaulus viviparus*	9,562	22560	0	0	0	95	0	3
Bmal	*Brugia malayi*	9,578	191089	0	0	0	110	0	3
Hduj	*Hypsibius dujardini*	10,223	50531	58	0	17(17)	50	0	86
Avit	*Acanthocheilonema viteae*	11,382	25808	0	0	0	41	0	3
Bxyl	*Bursaphelenchus xylophilus*	13,490	949830	0	0	0	21	0	14
Hbac	*Heterorhabditis bacteriophora*	14,630	33765	2	0	0	45	0	41
Mhap	*Meloidogyne hapla*	15,358	37608	0	0	0	65	0	62
Lloa	*Loa loa*	15,825	174388	0	0	0	134	0	0
Gpal	*Globodera pallida*	18,139	121687	0	0	0	70	3	92
Lsig	*Litomosoides sigmodontis*	20,478	45863	0	0	0	3	0	0
Acas	*Acanthamoeba castellanii*	30,713	564894	29	4	17(21)	23	58	46
Nve	*Nematostella vectensis*	33,008	472588	495	42	135(177)	908	24	1099
Dpul	*Daphnia pulex*	38,001	642089	301	58	125(183)	1012	455	1082
Crem	*Caenorhabditis remanei*	39,630	435512	16	3	8(11)	149	0	88
Tmur	*Trichuris muris*	50,312	400602	0	0	0	520	114	496
Cbre	*Caenorhabditis brenneri*	57,601	381961	0	0	0	136	0	78
Lgi	*Lottia gigantea*	80,338	1870055	333	17	138(155)	548	19	521
Pred	*Panagrellus redivivus*	97,659	270080	5	0	0	25	0	26
C11sp	*Caenorhabditis* sp11	119,280	20921866	0	0	0	27	0	14
Acal	*Aplysia californica*	214,107	917541	64	9	24(33)	202	26	538
Alyr	*Arabidopsis lyrata*	297,364	24464547	0	0	0	1209	1607	1454
Vcar	*Volvox carteri*	302,219	2599759	182	35	76(111)	515	435	618
Srat	*Strongyloides ratti*	1,047,729	4921549	0	0	0	18	0	40
briC	*Caenorhabditis briggsae*	9,034,972	17485439	33	8	17(25)	44	0	35
Cele	*Caenorhabditis elegans*	14,326,629	17493829	1	0	1(1)	28	2	11
Ath	*Arabidopsis thaliana*	17,095,393	23459830	0	0	0	252	374	433
Nvi	*Nasonia vitripennis*	59,454,730	48524378	101	9	38(47)	760	510	1428
		Total		2539	207	756(963)	11264	4102	12165

YR matches are shown, of which, the number of YREs that were classified based on their features (intact YREs) and their phylogenetic position (Partial YREs) is indicated. In addition, the counts of RT hits from BEL, *Copia* and *Gypsy* LTR elements are indicated for each species. Matches were found in all the species in at least one of the PSITBLASTN searches. The number of matches found in each species seems to be detached from the mean contig length or contig length at N50 in the species' genome assembly.

**Figure 3:**
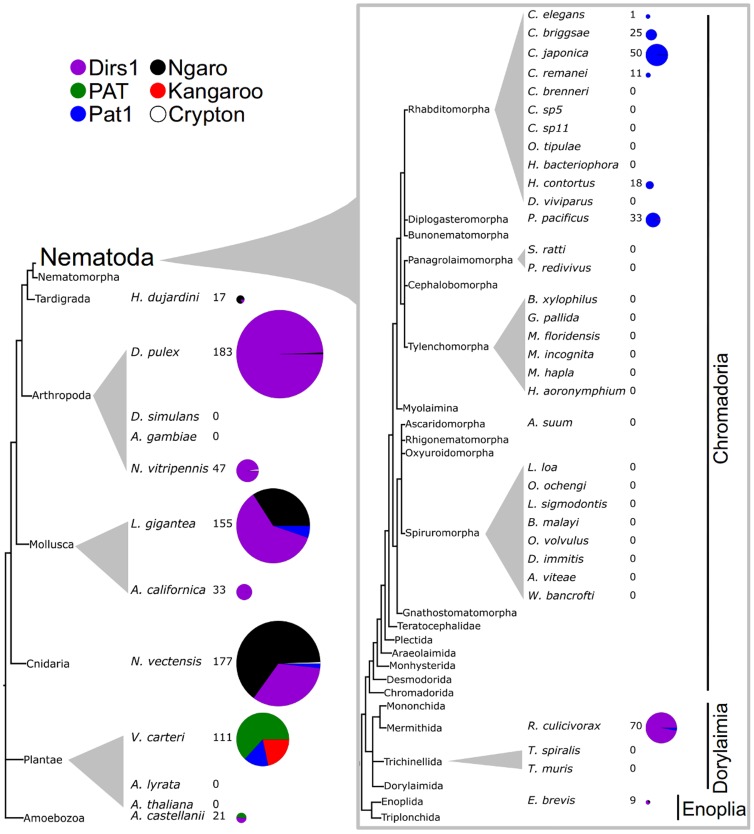
The distribution of YREs among Nematoda and outgroup species. The phylogenetic tree of Nematoda is based on De Ley and Blaxter [47] and Kiontke et al. [48]. Element types are colour coded. The phylogenetically classified YRE matches in each species are indicated. Pie-charts represent the proportion of each element type with their radii proportional to the number of phylogenetically classified YRE matches.

Our phylogenetic classification, based on YR domain sequences, included two steps (Figure 2A). In the first step, only complete elements, for which terminal repeats were identified, were considered, in order to delineate YRE clades. In the second step, all the putative YR matches were included, in order to classify partial elements based on their phylogenetic relationships with complete elements. After this phylogenetic classification (Figure S1), 963 elements were classified in 17 genomes (Figure 3).

To assess whether the genome assemblies used were of sufficient quality to permit YRE discovery, we also searched for RT domains from three LTR elements, *Gypsy*, *Copia* and BEL, reasoning that if we were unable to detect any of the abundant LTR class elements it was likely that the assembly was too poor. The N50 contig lengths of the assemblies (Table 1) did not correlate with the number of YRE matches (linear *R* ^2^ = 2*10^−3^, power *R* ^2^ = 8*10^−3^). A greater number of matches were found in outgroup taxa with larger genomes than Nematoda. No species had zero matches in all four searches (YRE plus three LTR searches). *Litomosoides sigmodontis* (Spiruromorpha;Spirurina;Rhabditida) had the lowest number of matches, including only three to BEL LTR retrotransposons, while *Oscheius tipulae* (Rhabditomorpha; Rhabditina; Rhabditida) had 10 or less matches in any searches. *Bursaphelenchus xylophilus* (Tylenchomorpha; Tylenchina;Rhabditida), *Caenorhabditis angaria* and *Caenorhabditis* sp. 11 (both Rhabditomorpha; Rhabditina; Rhabditida) had a maximum of 27 matches in any of the searches. For the remaining species, at least 40 matches were found in at least one of the searches. Given these findings, we are confident that cases where no YREs were found usually indicate a real absence, or extreme scarcity, of YREs in those species.

### Partition homogeneity test

Reciprocal AU-tests were conducted to test the phylogenetic homogeneity of the YR and RT domains, using datasets with identical element sampling. All the tests rejected the homogeneity of the two partitions, suggesting either a real difference in the phylogenetic history of the two markers, or low phylogenetic signal in one or both of the markers. Because the RT domain demonstrated a stronger phylogenetic signal, according to the sh-like node support values, we based our inference of the phylogenetic relationships between the different YRE lineages on phylogenetic analysis of the RT domains from complete YREs (Methods S1).

### Phylogenetics and distribution of YREs in the studied genomes

#### *Dirs1*-like elements

**Figure 4:**
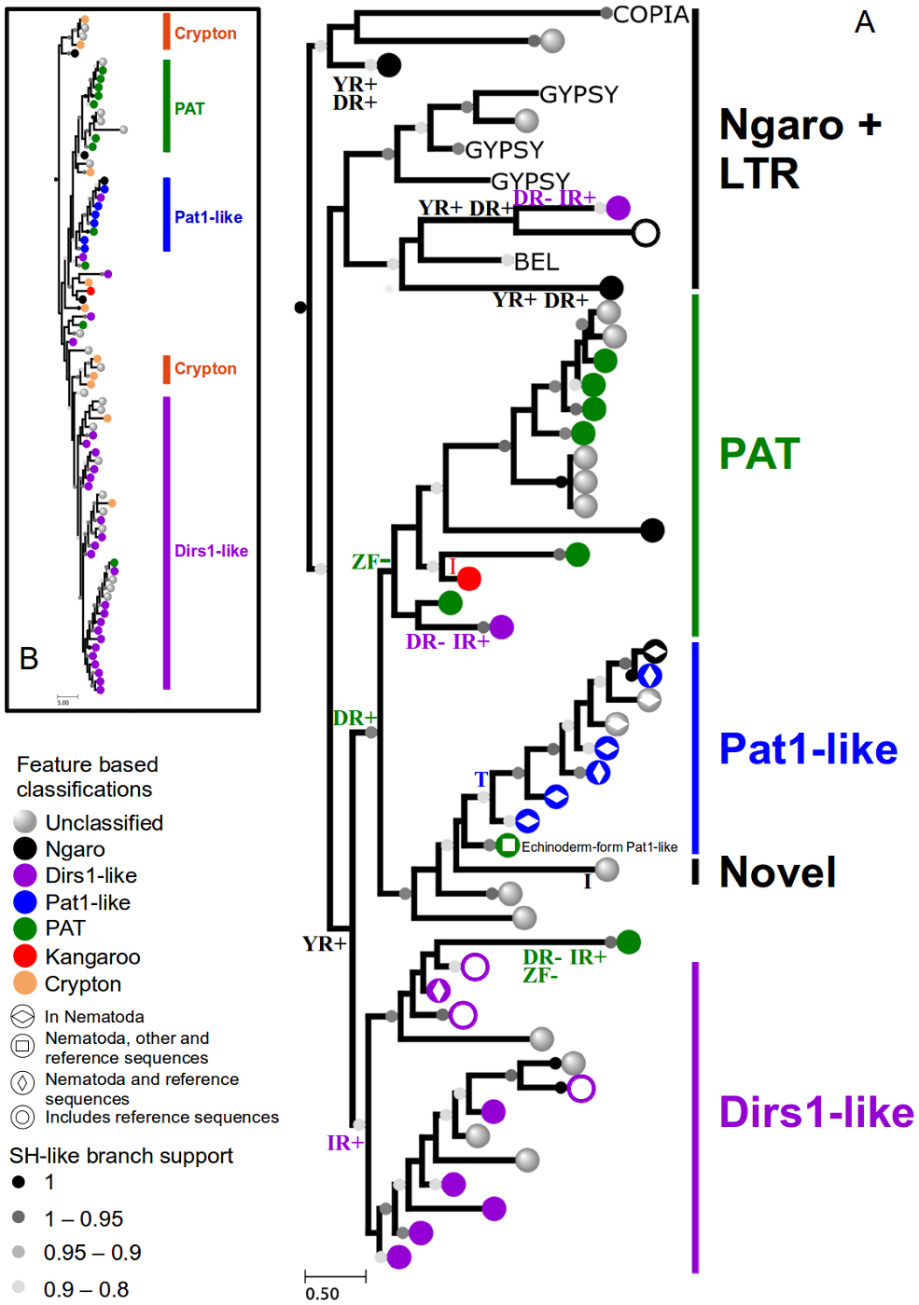
The phylogenetic relationships among YREs recovered from Nematoda and outgroup species. The phylogeny of the YREs was derived from analyses of the RT domains (A) and the YR domains (B). Character state changes of diagnostic YRE features are indicated as follows: YR: tyrosine recombinase domains; DR: split direct repeats; IR: inverted repeats; I: inversion of the YR domain; T: translocation of the internal repeats; zf: zinc finger in the Gag protein. sh - like branch supports are indicated at the base of nodes. Feature based classification, and the inclusion of reference sequences is indicated on each leaf. Where the leaves have a branch support symbol, these leaves are in fact collapsed clades.

More than half of the recovered YREs were phylogenetically classified as *Dirs1*-like (504 elements). *Dirs1*-like elements were recovered as one major lineage and two or more additional minor lineages in the RT (Figure 4A) and YR (Figure 4B) trees, respectively. One of the minor lineages clustered among PAT elements in both the YR and RT trees. The major lineage was paraphyletic (with respect to element classification by structural features; Figure 1) in both analyses and included a PAT group, which appeared to be misplaced in the RT tree (Figure 4A) due to its long branch.

Among the outgroup taxa, *Dirs1*-like elements were found in *Acanthamoeba castellanii*, Cnidaria, Mollusca and Arthropoda (Figure 3). In contrast to previous reports, *Dirs1*-like elements were also found in Nematoda. *Enoplus brevis* (Enoplida; Enoplia) and *Romanomermis culicivorax* (Mermitihida; Dorylaimia) had several *Dirs1*-like elements each (7 and 68, respectively). *E. brevis* elements were truncated and clustered with complete *Dirs1*-like elements from *Daphnia pulex* (Branchiopoda; Crustacea; Arthropoda; sh-like support of 0.96, Figure S1). The absence of intact elements in *E. brevis* is likely to be because of the short average contig length (447 bp) of this assembly. *R*. *culicivorax Dirs1*-like elements included five complete elements, which were most closely related to elements from the tardigrade *Hypsibius dujardini* (Parachela; Eutardigrada) (sh-like support >0.95, Figure S1).

In Chromadoria, a single partial *Dirs1*-like element was found in *P. pacificus*. It clustered with complete *Dirs1*-like elements from *R. culicivorax* (sh-like support >0.95, Figure S1). It had a long branch and no significant matches in the BLAST database and thus is marginal in terms of affirming YR ancestry. All the *Dirs1* instances found in Nematoda belong to the major *Dirs1*-like lineage (Figure 4).

#### PAT elements

A paraphyletic group of PAT elements, including *Pat1*, *Kangaroo*, one novel form (Figure 1G) and other PAT elements, which were not further classified, was recovered in the RT tree (Figure 4A). Its paraphyly was due to a single minor lineage of *Dirs1*-like elements, which clustered with the PAT lineages in both the RT and YR trees, and a single Ngaro lineage, which might be misplaced, considering its long branch. An additional PAT group clustered inside the *Dirs1* major linage. The *Pat1*-like lineage comprised 142 nematode-form *Pat1* elements (Figure 1C) and 27 echinoderm-form elements (Figure 1D). These 27 elements were classified as *Pat1*-like due to the presence of a zinc finger motif in the Gag sequence of some of them, in addition to their phylogenetic position. They clustered together with the echinoderm-form *Pat1*-like sequence from Retrobase (SpPat1). The *Pat1*-like elements of both forms (Figure 1C and 1D) formed a monophyletic clade in the RT tree (Figure 4A). In this clade, the echinoderm-form elements were early diverging. In the reduced YR tree (Figure 4B), the two forms were recovered as separate lineages.

*Kangaroo* elements from the alga *V. carteri* (24 elements) formed a single lineage within the PAT clade (Figure 4A). A PAT element in *D. pulex* was represented by four full and 6 truncated instances, clustered as a sister clade of the *Pat1*-like elements (labelled “novel”, Figure 4A). It was similar to PAT elements in structure, though possessing an inverted YR domain (Figure 1G). Unlike *Kangaroo* elements, which also have inverted YR domains, the novel element had internal repeats upstream to the 3′ terminal repeat and not between the MT and YR domains. The remaining unclassified PAT elements clustered paraphyletically in the RT tree (Figure 4A), but they clustered into three different lineages in the reduced YR tree (Figure 4B).

Echinoderm-form *Pat1*-like elements (Figure 1D) were found in the dorylaimid nematode *R. culicivorax*, the mollusc *Lottia gigantiea*, the cnidarian *Nematostella vecetensis* and the chlorophyte alga *V. carteri* (Figure 3). The *L. gigantiea* and *N. vecetensis Pat1*-like elements are most likely the same as the PAT elements reported in Piednoël et al. [12]. PAT elements lacking a Gag protein with a zinc finger motif were found only outside Metazoa. Lacking a zinc finger motif, these PAT elements could be considered to be *Toc3*-like (Figure 1F). However, many are partial elements from which Gag was not recovered. Thus, the precise identity of most PAT elements could not be determined.

The nematode-form *Pat1*-like elements (Figure 1C) were found in the nematode classes Dorylaimia and Chromadoria. In Chromadoria they were only detected in Rhabditomorpha and Diplogasteromorpha. The absence of *Pat1*-like elements from 23 out of the 29 sampled rhabditid species is surprising. Poor assembly quality cannot serve as the only explanation for this finding as several of the genomes lacking identified elements had good average contig length (Table 1). The absence of *Pat1* elements from *P. redivivus* was also unexpected, since this species is known to possess several *Pat1*-like elements [23] and a reciprocal blast approach was taken to confirm this finding. We queried the *P. redivivus* genome assembly using BLAST with the original *Pat1* sequence from *P. redivivus* (Genbank accession X60774). Twelve significant matches were found. For confirmation, these fragments were then used as queries to search the online NCBI BLAST database with default settings, detecting the original *Pat1* sequence (X60774) as a single hit. Since the matches were *Pat1* fragments that did not contain the YR ORF, they had not been recovered by our pipeline, and this lack of complete *Pat1* elements was likely due to incomplete assembly.

#### Non DIRS YREs

In the species surveyed we identified only a single Crypton element, in *N. vectensis*. This element has already been recorded in Repbase (locus Crypton-1_NV). An additional Crypton match in *Nasonia* was closely related to a previously identified element from oomycetes (locus CryptonF-6_PI in Repbase) and is likely to be a contamination. Using more lenient parameters, permitting larger clades with lower sh-like support to be included, increased the count of Crypton-like elements. However, this resulted in clades with simultaneous conflicting classifications. In addition, we identified three major lineages of Ngaro elements, including 182 instances that clustered with LTR elements. These lineages included the Ngaro reference sequences. An additional minor lineage, from *Caenorhabditis briggsae*, clustered closely with *Pat1*-like elements from the same species and showed minimal sequence divergence from them (Figure 4A). We suggest that this Ngaro lineage was a derived species-specific form of *Pat1* element that has lost its MT domain. Unlike Crypton elements, Ngaro elements were found in most of the animal phyla examined (Figure 3). Ngaro were abundant in the cnidarian *N. vectensis* (114 instances) and in the mollusc *L. gigantea* (53 instances). However, within Ecdysozoa, Ngaro counts were lower and ranged between 2 in *E. brevis* and 14 in *H. dujardini*.

### The evolution of YRE features

Based on the RT phylogeny, one of the possible most parsimonious scenarios for feature evolution is annotated in Figure 4. Under this hypothesis, the loss of the MT domain, the inversion of the YR domain, the formation of split direct repeats and of inverted repeats, and the loss of a zinc finger motif have each occurred more than once, independently, and both split direct repeats and inverted repeats must have been formed through multiple sequential inversions. Other possible scenarios would also require that several YRE features evolved in parallel. In addition, any possible scenario would be inconsistent with single step character changes between element types (Figure S2), given the phylogenetic analysis.

## Discussion

### Taxonomic representation in the study of TE

The distribution of transposable elements has been hypothesized to depend on a number of factors. Mating system, ploidy, zygosity, ecology and gene flow could all potentially influence the TE load and diversity in an organism, in addition to the constraints of its phylogenetic history [39]–[45]. Even within species, strains and populations can differ markedly in TE abundance [25]. Therefore, when studying the distribution of TEs, it is unlikely that a single or a few species would accurately represent a whole phylum, especially a phylum as species rich and diverse as Nematoda.

Piednoël et al. [12] surveyed *Dirs1*-like YREs in a wide range of eukaryotes in order to understand the distribution of this element. Although they analysed 274 genome assemblies, only two nematode genomes were available, and these were from two closely related rhabditid superorders, Rhabditomorpha (*C. elegans*) and Diplogasteromorpha (*P. pacificus*). Neither species contained *Dirs1*-like sequences, leading to the conclusion that these elements were absent from nematodes as a whole. In the current study, however, thanks to the wider taxonomic representation that is now available, we have identified *Dirs1*-like sequences in at least two out of the three nematode subclasses.

In addition, since many of the assemblies we screened were drafts and thus highly fragmented representations of the original genomes (the shortest average contig length was 411 bp in *Howardula aoronymphium*), we employed a search strategy that did not require the presence of complete YRE sequences, which may be as long as 6,000 bp [12]. This approach, together with the classification of complete elements based on their structure, and the phylogenetic analysis of both complete and truncated elements, allowed us to recover and classify about 700 truncated YREs. To illustrate the power of this approach, while *E. brevis* had an average contig length of 477 bp, we recovered nine elements that were classified based on their phylogenetic relationship with reference sequences, which would have otherwise been missed. These results emphasize the importance of dense taxonomic sampling and of the inclusion of truncated elements in surveys of element diversity and distribution. Still, the failure to identify the expected *Pat1* elements in *P. redivivus* illustrates that the quantification and identification of TEs cannot be complete while focusing solely on protein domains and genome assemblies.

### YRE content in Nematoda has undergone a shift

Based on our findings, Nematoda has undergone a substantial change in the composition and numbers of YREs (Figure 3). The YRE content of the enoplid and dorylaimid species examined was more similar to that of outgroup taxa in *Dirs1* proportions than the YRE content of the rhabditid species. *Dirs1*-like elements, relatively abundant in some outgroups, were found in *E. brevis* and *R. culicivorax* but were sparse in Rhabditida. The only potential *Dirs1*-like element found in Rhabditida was probably misclassified or a result of contamination, and *Dirs1*-like elements may be absent from Rhabditida altogether. In addition, the echinoderm-form *Pat1*-like element is found in *R. culicivorax* but not in other nematodes. It will be very informative to sample species from additional chromadorid superorders to identify the mode and tempo of this loss.

### The evolution of PAT elements

The known distribution of the *Pat1* group of elements in Metazoa has been puzzling. *Pat1* elements were previously found only in Nematoda (Ecdysozoa) [11], [12], [23] and Echinodermata (Deuterostomia) and the elements from these phyla have distinctly different feature organisations [11], [12], [19], [23]. Piednoël et al. [12] were unable to classify the PAT elements from Cnidaria and Mollusca as *Pat1*. Consequently, the known distribution of the *Pat1* group of elements in Metazoa was puzzling. Here, through the phylogenetic classification of truncated elements, we identified the PAT elements in Mollusca and Cnidaria as *Pat1*-like, suggesting that these elements, though rare in general, are found in all three branches of Bilateria, and in non-bilaterian Metazoa. Surprisingly, the *Pat1*-like element that was found in the nematode *R. culicivorax* has an echinoderm-form structural arrangement rather than the nematode-form. In addition, *Pat1*-like elements from Nematoda and from Echinodermata form sister clades in the RT tree (Figure 4A). Thus, the nematode-form *Pat1*-like element is not an isolated element with an unknown origin, but rather a taxon specific clade of a more widespread *Pat1* element family, and we suggest that there exists a greater diversity of these elements yet to be discovered.

### Homoplasy in YRE structural features and the need for phylogenetics

YREs have been suggested to have emerged from a composite ancestor combining an LTR element with a Crypton, as both Cryptons and LTRs are considered to be more ancient than YREs based on their distribution [32]. It is not clear, however, whether a single or several independent events of recombination are at the base of YRE retroelements. Our results support at least two origins for YRE retroposons: at least one for Ngaro elements and one for DIRS elements. As a consequence, split direct repeats must have evolved more than once, independently, resulting in homoplasious similarity. This result is in accordance with the phylogenetic tree presented in Goodwin and Poulter [19]. While Goodwin and Poulter [19] found that PAT and *Dirs1*-like elements form a single clade each, we observed a paraphyletic, or possibly polyphyletic *Dirs1* group. Since this was observed in both the RT and YR trees (Figure 4), thiscould either mean that PAT elements evolved from *Dirs1* or that a *Dirs1*-like element evolved twice independently. It is worth noting that the formation of inverted repeats from split direct repeats is a complex process that would require some intermediate forms. However, these forms are not observed, possibly due their inviability.

Another homoplasious similarity between polyphyletic element lineages was observed in Ngaro and a derived lineage of *Pat1*-like elements in *C. briggsae*, both of which lack a MT domain. In addition, a derived PAT element in *D. pulex* had homoplasious similarity (an inverted YR domain) to *Kangaroo* from *V. carteri*. Also, we infer that the loss of a zinc finger motif from the Gag protein must have occurred independently multiple times. Taking these observations together, homoplasy in element features is a strong theme in the evolution of YREs. This strongly suggests that it is impractical to use structural characteristics as the sole descriptors for element classification, and that incorporating an explicitly phylogenetic basis for classification will produce more biologically meaningful inferences.

## Conclusions

In this study we utilised a large number of nematode genome assemblies to characterize the YRE content in Nematoda. We showed that the YRE content across the phylum is much more diverse than suggested by the analysis of a few model species. The inclusion of truncated elements filled the gaps in the extant diversity of both *Dirs1*-like and *Pat1*-like elements, both of which are more widely distributed than originally thought. Our results strongly support a previous call [46] to classify transposable elements based on phylogenetic relationships rather than the features they contain or lack, thus conforming to a systematic approach to classification.

## Supporting Information

Figure S1 **The phylogenetic classification of the recovered YREs.** This phylogeny was reconstructed using only YR sequences from elements with defined borders (also available as Figure 2B), with a midpoint root (white background). Clades from the full YR tree (in grey) are presented next to reduced tree clades with which they share leaves. Large font black leaves are shared between the full and reduced YR trees. Large font green leaves are additional reference sequences. Small font leaves from the full tree (in grey) were added to the leaf count of the corresponding reduced tree clade. Only full tree clades with sh-like support >0.9 were considered. Full tree clades that included long branches were removed if they had sh-like support <0.95. The branch-length cutoff was four times the median branch-length of the clade. Leaf names include the species code (as in Tables 1 and S1), a unique number and the feature based classification. The unique number is the start position of the YR domain on its contig. table.out files in the pipeline results folder (http://dx.doi.org/10.6084/m9.figshare.1004150) provide access to the complete element information using the species code and the unique number. The unique number provides access to the element's diagram in the same folder.
(PDF)

Figure S2 **Hypothetical single step transitions between different YRE retrotransposon types.** A flow chart depicting all the possible single step transitions between YRE retrotransposon types, using Ngaro as the ancestral form. Dirs1-like elements cannot be created from other element types in a single step. This scenario is not supported by the phylogenetic analysis (Figure 2).
(PDF)

Table S1 **Source of genomic data.** Abbreviation, taxonomy and genome assembly information of the species studied.
(CSV)

Methods S1 **The IPython notebook with which all the analyses related to this study were conducted is provided here as a static html file.** It includes all the scripts along with detailed information. The executable IPython notebook is available in the github repository (http://dx.doi.org/10.6084/m9.figshare.1004150) along with the input and output files, except for the genome assemblies, which were very large. The genome assemblies can be accessed via links in Table S1 or in the iPython notebook.
(HTML)
