# Supplementary material for: The Evolution of Tyrosine-Recombinase Elements in Nematoda
Source: PLoS One. 2014 Sep 8;9(9):e106630. doi: 10.1371/journal.pone.0106630 (PMC4157794; doi:10.1371/journal.pone.0106630)
Supplement: Figure S2 — Hypothetical single step transitions between different YRE retrotransposon types. A flow chart depicting all the possible single step transitions between YRE retrotransposon types, using Ngaro as the ancestral form. Dirs1-like elements cannot be created from other element types in a single step. This scenario is not supported by the phylogenetic analysis (Figure 2). (PDF) [file pone.0106630.s002.pdf]

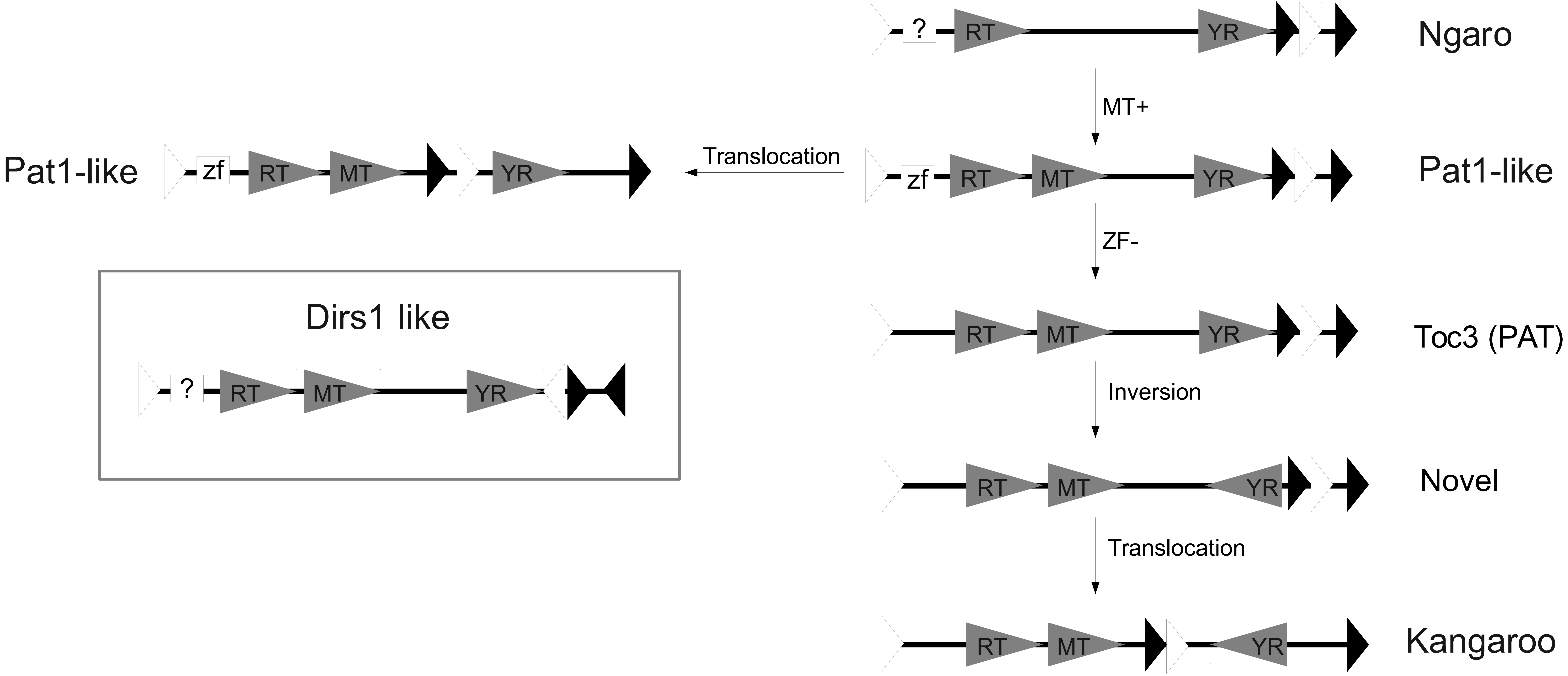
Ngaro
?
RT
YR
MT+
Pat1-like
zf
RT
MT
YR
Translocation
Pat1-like
zf
RT
MT
YR
ZF-
Dirs1 like
?
RT
MT
YR
Toc3 (PAT)
RT
MT
YR
Inversion
Novel
RT
MT
YR
Translocation
Kangaroo
RT
MT
YR
