# Supplementary material for: The Evolution of Tyrosine-Recombinase Elements in Nematoda
Source: PLoS One. 2014 Sep 8;9(9):e106630. doi: 10.1371/journal.pone.0106630 (PMC4157794; doi:10.1371/journal.pone.0106630)
Supplement: Methods S1 — The IPython notebook with which all the analyses related to this study were conducted is provided here as a static html file. It includes all the scripts along with detailed information. The executable IPython notebook is available in the github repository (http://dx.doi.org/10.6084/m9.figshare.1004150) along with the input and output files, except for the genome assemblies, which were very large. The genome assemblies can be accessed via links in Table S1 or in the iPython notebook. (HTML) [file pone.0106630.s004.html]

[Szitenberg et al. 2014]


# Analysis workflow

In [32]:

```
from IPython.core.display import Image 
Image(filename='Figure S1.png', width=500)
```

Out[32]:

# Dependencies

**Python**  
Some python modules have their own dependancies, see links:  
ete2  
pycogent  
Biopython  
Dendropy  
psiblast\_ugene\_TREs\_search\_pipline.py  
  
 **Perl**

# Required input file

**psiblast\_ugene\_TREs\_search\_pipline.py** - the pipline  
**Fitch2013\_Adapted\_DeLey2006.tre** - A cartoon Nematoda tree to draw results  
**nematode\_species.csv** - code name and taxonomy of the studied species  
**renamed\_nodes.txt** - corrects a few pipeline derived classifications blased on preliminary results  
**GG.msa** - query for building a Gag domain sequence model  
**one\_seq.txt** - subject file for the construction of the Gag model  
**query\_model\_seeds/YR.msa** - query for building a YR domain sequence model  
**query\_model\_seeds/RT.msa** - query for building a RT domain sequence model  
**query\_model\_seeds/MT.msa** - query for building a MT domain sequence model  
**query\_model\_seeds/All\_proteins.fasta** - all the proteins from TEs from RepBase, Retrobase and Pat1 elements to be used for building the protein sequence model  
**LTR\_sequences/RT\_outgroup.fas** - to be added to the RT dataset for phylogenetic analysis

# Make folders

In [1]:

```
import os

for folder in ('comparable_trees',
               'GG',
               'RT',
               'YR_tree',
               'psiblast_ugene_RESULTS',
               'query_model_files'):
    if not os.path.exists(folder):
        os.makedirs(folder)
    else:
        raise Exception('folder ' + folder + ' already exists')
```

# Make query models from query seed alignments

I am using seed query alignments to query all the DIRS, Ngaro and crypton proteins from repbase, retrobase and some additional pat elements and get an extended MSA file to serve as a basis for query models.  
The seed alignments are as follows:   
 For RT, MT and YR I am using the query alignment from Piednoël et al. (2012). For GAG I am using some proteins from Repase and retrobase.

In [2]:

```
from Bio.Blast.Applications import NcbipsiblastCommandline
import os

files = os.listdir('query_model_seeds')
MSAs = []

for name in files:
    if '.msa' in name:
        MSAs.append(name)

os.system('makeblastdb -in query_model_seeds/All_proteins.fasta -dbtype prot')        
        
for msa in MSAs:
    ckp = msa.split('.')[0] + '.ckp'
    psiblast_cline = NcbipsiblastCommandline(in_msa= 'query_model_seeds/' + msa,
                                             db='query_model_seeds/All_proteins.fasta',
                                             evalue=0.00000000001,
                                             num_iterations = 10,
                                             out_pssm= 'query_model_files/' + ckp)
    stdout, stderr = psiblast_cline()
```

# Run PSIBLAST - UGENE pipline

The following cell contains a scripts that runs psiblast\_ugene\_TREs\_search\_pipline.py for each of the assemblies. The script itself is an external file that is provided in the folder and is not writen in this notebook. The pipeline first searches for YR domains using PSITBLASTN. Then it extends the hits by 10 kbp in each direction and searhces RT and MT domains as well as direct and inverted repeats on the resulting fragments using UGENE. The annotated fragments are searched for a distinct element structure. Following is a short discription of the used parameters and the output files for each assembly.

## Commandline arguments

>> /path/to/folder/psiblast\_ugene\_TREs\_search\_pipline.py [species\_code\_name][path\_to\_assembly\_fasta] [assembly\_fasta\_file][comment] [path\_to\_results\_folder][path\_to\_query\_model\_files]

## Additional parameters that can be managed from within psiblast\_ugene\_TREs\_search\_pipline.py

1. **extend: the fragment length in base-pairs from each side of the YR hit, that will be searched for MT and RT domains, as well as repeat sequences (current setting 10,000, i.e., the fragment will be 20,000 bp long plus the length of the YR hit, unless the contig is shorter than that).**  
    This value was chosen based on the assumption that DIRS elements are less than 6 kb in length (Piednoël et al. BMC Genomics 2011, 12:621). An artifact of this may be the inclussion of protein domains from different elements as one element. To avoid this, only RT and MT domains that are circumscribe between element related repeats together with a YR domain were included in the final dataset.
2. **intron: the length of allowed intron within a domain. Two hits of the same domains will be concatenated if the gap between them is less than the intron maximum length (current setting 100).**  
    Concatenated sequences aligned nicely with other elements.
3. **e\_vlaue: the psitblastn e-value cutoff (current setting 0.01).**  
    This value was chosen following (Piednoël et al. BMC Genomics 2011, 12:621). In addition, further support for the homology of the hits is obtained when the structure of a whole element is detected.
4. **maxdist: the maximum distance between repeat pair partners (ugene, current setting 20,000).**
5. **mindist: the minimum distance between repeat pair partners (ugene, current setting 0).**  
    Since the fragment are about 20,000 bp long, the maxdist and mindist are non-restrictive and are making no assumptions.
6. **minlength: the minimal repeat length (ugene, current setting 20).**  
    Based on the results of (Piednoël et al. BMC Genomics 2011, 12:621), the shortest repeat sequence is a central repeat sequence 23 bp long. Central repeats of such length are always identical to the part of the terminal repeat they align with.
UGENE parameters may determine whether a sturcture is identified or not for a minority of the identified elements.

## Pipeline output files per assembly

 The results folder contain a subfolder for each assembly. These subflders are named with the assembly code as listed in the code cell bellow. Each subfolder contains:

1. **table.out:** A table with the following info for each identified TRE: Fragment(species:contig:YR\_start\_on\_contig), Structure\_Indication, start\_on\_contig, end\_on\_contig, Contig\_length, Fragment\_length, YR\_evalue, RT\_evalue and MT\_evalue.
2. **YR\_fragments.embl:** YR fragment sequences in embl format. The features include all the protein domains and repeat sequences found. The TE feature provide the element's approximate borders. Since only identical repeats are searched for, the repeats borders are approximate. The locations provided are fuzzy, although they are formated as precise.
3. **YR\_fragments.fas:** YR fragment sequences in fasta format.
4. **.xml:** The PSIBLAST results. Three .xml files exist, one for each domain (YR, RT and MT). The YR results provide the location of the YR domain on the contig and the RT and MT results provide the location on the YR fragment (ie, the YR extanded hit)
5. **\_aa.fas:**  Fasta file containing the protein domains aa sequeces. There are three files, one for each domain (YR, RT and MT)
6. **\_direct.out and \_inverted.out:** UGENE results in GB format.
7. **The fragments\_diagrams and TEs\_diagrams folders:** Contain diagrams of all the features found on each fragment or only the features between the element borders, respectively

## TE features used for classification by the pipeline

  

## Links to the genome assembliy files analysed

1. **Bxyl** Bursaphelenchus xylophilus ftp://ftp.sanger.ac.uk/pub/pathogens/Bursaphelenchus/xylophilus/Assembly-v1.2/BurXv1.2.supercontigs.fa.gz
2. **Asuu** Ascaris suum ftp://ftp.wormbase.org/pub/wormbase/species/a\_suum/sequence/genomic/a\_suum.PRJNA62057.WS238.genomic.fa.gz
3. **Wban** Wuchereria bancrofti http://www.broadinstitute.org/annotation/genome/filarial\_worms/download/?sp=EASupercontigsFasta&sp=SWuchereria\_bancrofti\_V1&sp=S.zip
4. **Ppac** Pristionchus pacificus ftp://ftp.wormbase.org/pub/wormbase/species/p\_pacificus/sequence/genomic/p\_pacificus.PRJNA12644.WS238.genomic.fa.gz
5. **Ebre** Enoplus brevis Genepool, in annotation
6. **Rcul** Romanomermis culicivorax Genepool, in annotation
7. **Dviv** Dictyocaulus viviparus Genepool, in annotation
8. **Pred** Panagrellus redivivus http://www.ncbi.nlm.nih.gov/nuccore?term=KB454917:KB455574[PACC]
9. **Cang** Caenorhabditis angaria ftp://ftp.wormbase.org/pub/wormbase/species/c\_angaria/sequence/genomic/c\_angaria.PRJNA51225.WS238.genomic.fa.gz
10. **Cbre** Caenorhabditis brenneriftp://ftp.wormbase.org/pub/wormbase/species/c\_brenneri/sequence/genomic/c\_brenneri.PRJNA20035.WS238.genomic.fa.gz
11. **briC** Caenorhabditis briggsae ftp://ftp.wormbase.org/pub/wormbase/species/c\_briggsae/sequence/genomic/c\_briggsae.PRJNA10731.WS238.genomic.fa.gz
12. **Cele** Caenorhabditis elegans ftp://ftp.wormbase.org/pub/wormbase/species/c\_elegans/sequence/genomic/c\_elegans.WS235.genomic.fa.gz
13. **Cjap** Caenorhabditis japonica ftp://ftp.wormbase.org/pub/wormbase/species/c\_japonica/sequence/genomic/c\_japonica.PRJNA12591.WS238.genomic.fa.gz
14. **Crem** Caenorhabditis remanei ftp://ftp.wormbase.org/pub/wormbase/species/c\_remanei/sequence/genomic/c\_remanei.PRJNA53967.WS238.genomic.fa.gz
15. **C5sp** Caenorhabditis sp. 5 ftp://ftp.wormbase.org/pub/wormbase/releases/WS230/species/c\_sp5/c\_sp5.WS230.genomic.fa.gz
16. **C11sp** Caenorhabditis sp11 http://genome.wustl.edu/pub/organism/Invertebrates/Caenorhabditis\_sp11\_JU1373/assembly/Caenorhabditis\_sp11\_JU1373-3.0.1/output/supercontigs.fa.gz
17. **Hbac** Heterorhabditis bacteriophora http://genome.wustl.edu/pub/organism/Invertebrates/Heterorhabditis\_bacteriophora/assembly/Heterorhabditis\_bacteriophora-1.2.1/output/contigs.fa.gz
18. **Otip** Oscheius tipulae http://nematodes.org/downloads/959nematodegenomes/blast/db/Oscheius\_tipulae\_clc3\_1.fna
19. **Srat** Strongyloides ratti ftp://ftp.sanger.ac.uk/pub/pathogens/Strongyloides/ratti/version\_4/Sratti\_v4.genome.fa
20. **Avit** Acanthocheilonema viteae http://nematodes.org/downloads/959nematodegenomes/blast/db2/Acanthocheilonema\_viteae\_v1.0.fna.gz
21. **Bmal** Brugia malayi ftp://ftp.wormbase.org/pub/wormbase/species/b\_malayi/sequence/genomic/b\_malayi.PRJNA10729.WS238.genomic.fa.gz
22. **Dimm** Dirofilaria immitis http://nematodes.org/downloads/959nematodegenomes/blast/db2/Dirofilaria\_immitis\_2.2.fna.gz
23. **Lsig** Litomosoides sigmodontis http://badger.bio.ed.ac.uk/filarial/fileDownload/zip\_download?fileName=Litomosoides\_sigmodontis\_2.1.fna
24. **Lloa** Loa loa http://www.broadinstitute.org/annotation/genome/filarial\_worms/download/?sp=EASupercontigsFasta&sp=SLoa\_loa\_V3&sp=S.zip
25. **Ooch** Onchocerca ochengi http://www.nematodes.org/downloads/959nematodegenomes/blast/db2/Onchocerca\_ochengi\_nuclear\_assembly\_nOo.2.0.fna.gz
26. **Ovol** Onchocerca volvulus http://www.broadinstitute.org/annotation/genome/filarial\_worms/download/?sp=EASupercontigsFasta&sp=SOnchocerca\_volvulus\_V1&sp=S.zip
27. **Hcon** Haemonchus contortus ftp://ftp.wormbase.org/pub/wormbase/species/h\_contortus/sequence/genomic/h\_contortus.PRJEB506.WS238.genomic.fa.gz
28. **Tspi** Trichinella spiralis ftp://ftp.wormbase.org/pub/wormbase/species/t\_spiralis/sequence/genomic/t\_spiralis.PRJNA12603.WS238.genomic.fa.gz
29. **Tmur** Trichuris muris ftp://ftp.sanger.ac.uk/pub/pathogens/Trichuris/muris/genome/version\_2b/Tmuris\_v2b.genome\_scaffolds.fa

In [3]:

```
import os

genomes_path = '/media/amir/DATA/work/Genomes/'

genomes = {
            'PAT_DNA_seqs_nematoda.fas': 'PAT',
            'DIRS_crypton_autonomous.fasta': 'BAS',
            'RetroBase.fas': 'RET',
            'meloidogyne.velvet.fa': 'Mflo',
            'dictyocaulus.velvet.fa': 'Dviv',
            'enoplus.clc.fa': 'Ebre',
            'romanomermis.clc.fa': 'Rcul',
            'Acanthocheilonema_viteae_v1.0.fa': 'Avit',
            'a_suum.PRJNA62057.WS238.genomic.fa': 'Asuu',
            'BurXv1.2.supercontigs.fa': 'Bxyl',
            'Heterorhabditis_bacteriophora-1.2.1.contigs.fa': 'Hbac',
            'Howardula_aoronymphium_clc_1.fa': 'Haor',
            'h_contortus.PRJEB506.WS238.genomic.fa': 'Hcon',
            'Sratti_v4.genome.fa': 'Srat',
            'c_briggsae.PRJNA10731.WS238.genomic.fa': 'briC',
            'c_elegans.WS235.genomic.fa': 'Cele',
            'c_japonica.PRJNA12591.WS238.genomic.fa': 'Cjap',
            'b_malayi.PRJNA10729.WS238.genomic.fa': 'Bmal',
            'c_brenneri.PRJNA20035.WS238.genomic.fa': 'Cbre',
            'c_remanei.PRJNA53967.WS238.genomic.fa': 'Crem',
            'Gpal.genome.30052012.scaffolds.fa': 'Gpal',
            'Litomosoides_sigmodontis_2.1.fna': 'Lsig',
            'p_pacificus.PRJNA12644.WS238.genomic.fa': 'Ppac',
            'loa_loa_v3_3_supercontigs.fasta': 'Lloa',
            'm_hapla.PRJNA29083.WS238.genomic.fa': 'Mhap',
            'm_incognita.PRJEA28837.WS238.genomic.fa': 'Minc',
            'c_sp5.WS230.genomic.fa': 'C5sp',
            'Dirofilaria_immitis_2.2.fa': 'Dimm',
            'Onchocerca_ochengi_nuclear_assembly_nOo.2.0.fna': 'Ooch',
            'onchocerca_volvulus_1_supercontigs.fasta': 'Ovol',
            'Oscheius_tipulae_clc3_1.fa': 'Otip',
            'Panagrellus_redivivus.KB454917-KB455574.fasta': 'Pred',
            'Caenorhabditis_sp11_JU1373-3.0.1.supercontigs.fa': 'C11sp',
            'c_angaria.PRJNA51225.WS238.genomic.fa': 'Cang',
            'Tmuris_v2b.genome_scaffolds.fa': 'Tmur',
            't_spiralis.PRJNA12603.WS238.genomic.fa': 'Tspi',
            'wuchereria_bancrofti_1_supercontigs.fasta': 'Wban',
            'Acas20100210.contigs.agp.linear.fa': 'Acas',
            'Anopheles_gambiae._S-4.2.supercontigs.fa': 'Agam',
            'A_californica_v0.assembly.fasta': 'Acal',
            'Daphnia_pulex.fasta': 'Dpul',
            'Drosophila_simulans_w501-1.1.contigs.fa': 'Dsim',
            'Lotgi1_assembly_scaffolds.fasta': 'Lgi',
            'Nvit_2.0.linear.fa': 'Nvi',
            'Nemve1.fasta': 'Nve',
            'Ath_TAIR10.fas': 'Ath',
            'Vcarteri_199.fa': 'Vcar',
            'Alyrata_107.fa': 'Alyr',
            'Hypsibius_dujardini_nHd.2.3.abv500.fna': 'Hduj',
            }
for genome_name in genomes.keys():
    os.system('python psiblast_ugene_TREs_search_pipline.py ' 
              + genomes[genome_name] + ' ' + genomes_path + ' ' + genome_name + ' ' + genome_name 
              + ' psiblast_ugene_RESULTS/ ' 
              + 'query_model_files/')
```

# Get all the TE DNA sequences

In order to look for zinc fingers in gag proteins

In [4]:

```
%%script perl
#!/usr/bin/perl

use strict;
use warnings;
use Bio::SeqIO;


my $folder = 'psiblast_ugene_RESULTS/';
my $PT = "TE_SEQS";
my $outfile = $folder.$PT.".fasta";
open OUT, ">$outfile";
my $x = $folder.'*/*_YR_fragments.embl';

for my $d (< $x >){
	$d =~ m/.+\/([^_]+)_YR_fragments.embl/;
	my $name = $1;
	my $format = "embl";
	my $inseq = Bio::SeqIO->new(-file => $d, -format => $format);

	if (-e($d)){
		while (my $seq = $inseq->next_seq){
			for my $feature($seq -> get_SeqFeatures){
				if ($feature -> primary_tag eq "TE"){
					my $cds = $feature -> spliced_seq -> seq;
			        for my $tag ($feature -> get_all_tags){
			        	if ($tag eq "element"){
				            my $value;
					        for my $i ($feature -> get_tag_values($tag)){
						        $value = $i;
					        }
					        print OUT ">", $name, '_', $seq -> accession, '_', $value ,"\n",$cds,"\n";
				        }
			        }
		        }
	        }
        }
    }
}

close OUT;
```

# Find zink fingers in GAG proteins

In [5]:

```
import os, sys, re
from Bio import SeqIO, SearchIO
from Bio.SeqIO import SeqRecord
from Bio.Seq import Seq
from Bio.Alphabet import IUPAC

from Bio.Blast.Applications import NcbitblastnCommandline, NcbipsiblastCommandline

#first search for GAG proteins with a query adapted for PAT elements

folder = 'psiblast_ugene_RESULTS/'
path = './'

os.system('makeblastdb -in ' + folder + 'TE_SEQS.fasta -dbtype nucl')

#make PAT adapted GAG model to search for zinc-fingers in PAT elements
psiblast_cline = NcbipsiblastCommandline(in_msa= './GG.msa',
                                             subject='./one_seq.txt',
                                             out_pssm= 'GG/GG.ckp')
stdout, stderr = psiblast_cline()

zf_count = 0
iterate = 1

seen_zf = []
while iterate == 1:
    
    #search TE DNA for GAG AA
    psiblast_cline = NcbitblastnCommandline(in_pssm='GG/GG.ckp', db=folder + 'TE_SEQS.fasta', evalue=0.0001, outfmt=5, out= path + 'GG/psiblast.out')
    stdout, stderr = psiblast_cline()
    
    #make GAG AA fasta file
    GG_filename = path + 'GG/GG.fas'
    GG_blast_results = SearchIO.parse(path + 'GG/psiblast.out', 'blast-xml')
    GG_seqs = []
    for hit in GG_blast_results:
        for hsp in hit.hsps:
            GG_seqs.append(hsp.hit)
    
    #make singular names and remove gaps
    singular = 0
    for record in GG_seqs:
        record.seq = Seq(re.sub('-','',str(record.seq)), IUPAC.protein)
        record.id = str(singular) + '_' + record.id 
        singular += 1
    SeqIO.write(GG_seqs, GG_filename, "fasta")
    
    #Search for zinc finger motifs
    
    ZF_pattrns = {
                  'dirs1': ['C[A-Z]{3}C[A-Z]{9}H[A-Z]{2}C[A-Z]{2}C[A-Z]{4}H[A-Z]{9}C[A-Z]HC',
                            'C[A-Z]{3}C[A-Z]{9}H[A-Z]{2}C[A-Z]{2}C[A-Z]{4}H[A-Z]{10}C[A-Z]HC',
                            'C[A-Z]{2}CC[A-Z]{6}H[A-Z]{4}C[A-Z]{2}C',
                            'C[A-Z]{2}C[A-Z]{9}H[A-Z]{2}C[A-Z]{2}C[A-Z]{4}H[A-Z]{4}C[A-Z]{2}C'
                            ],
                  'pat1_Ngaro':  ['C[A-Z]{2}C[A-Z]{4}H[A-Z]{4}C'],
                  'Ngaro': ['C[A-Z]{7}C[A-Z]{2}H[A-Z]C',
                            'C[A-Z]{2}C[A-Z]{3}H[A-Z]{4}C',
                            'C[A-Z]{7}C[A-Z]{5}C[A-Z]{3}H',
                            'C[A-Z]{8}C[A-Z]{5}C[A-Z]{3}H',
                            'C[A-Z]{2}C[A-Z]{3}H[A-Z]{6}C',
                            'C[A-Z]{8}C[A-Z]{4}H[A-Z]{3}H',
                            'C[A-Z]{2}C[A-Z]{4}H[A-Z]{4}H',
                            'C[A-Z]{11}C[A-Z]H[A-Z]{6}H',
                            'C[A-Z]{2}C[A-Z]{4}H[A-Z]{6}C',
                            'C[A-Z]{13}C[A-Z]{5}C[A-Z]{5}H',
                           ]                      
                  }
    
    
    
    new_zf_count = 0
    ZF_hits = {}
    records = list(SeqIO.parse(GG_filename, 'fasta'))
    for record in records:
        ZF_hits[record.id] = {}
        for element in ZF_pattrns.keys():
            ZF_hits[record.id][element] = 0
            for pattern in ZF_pattrns[element]:
                hits = re.findall(pattern, str(record.seq), re.IGNORECASE)
                ZF_hits[record.id][element] += len(hits)
            if ZF_hits[record.id][element] > 0:
                new_zf_count += 1             
                line = (
                        record.id + '	' 
                        + str(ZF_hits[record.id][element]) + '	' 
                        + element
                       )
                line_parts = line.split('_')
                line = '_'.join(line_parts[1:])            
                if not line in seen_zf:
                    seen_zf.append(line)

    if new_zf_count == zf_count:
        iterate = 0
    zf_count = new_zf_count
    
    #align hit GAG AA sequences
    os.system('mafft ' + GG_filename + ' > ' + GG_filename +'.aln')
    
    #convert to blast in_msa format
    SeqIO.convert(GG_filename +'.aln', 'fasta', GG_filename +'.msa', 'clustal')
    IN = open(GG_filename +'.msa', 'r').readlines()
    OUT = open(GG_filename +'.msa', 'wt')
    for line in IN[3:]:
        OUT.write(line)
    OUT.close()

    #make new ckp file from new alignment
    psiblast_cline = NcbipsiblastCommandline(in_msa = GG_filename +'.msa',
                                             subject = './one_seq.txt',
                                             out_pssm = './GG/GG.ckp')
    stdout, stderr = psiblast_cline()


for line in seen_zf:
    print line
```

```
Lgi_150_453169_PAT	1	pat1_Ngaro
Lgi_59_1690142_DIRS	1	pat1_Ngaro
Lgi_59_1685024_DIRS	1	pat1_Ngaro
Lgi_246_5989_DIRS	1	pat1_Ngaro
Lgi_246_5423_DIRS	1	pat1_Ngaro
Nve_318_58153_PAT	1	pat1_Ngaro
Nve_318_58227_PAT	1	pat1_Ngaro
Hcon_0059030_17723_pat1-like	1	pat1_Ngaro
Hcon_0059030_17196_pat1-like	1	pat1_Ngaro
Rcul_11614_5929_DIRS	1	pat1_Ngaro
Rcul_65775_27385_DIRS	1	pat1_Ngaro
```

```
/usr/local/lib/python2.7/dist-packages/Bio/SearchIO/__init__.py:213: BiopythonExperimentalWarning: Bio.SearchIO is an experimental submodule which may undergo significant changes prior to its future official release.
  BiopythonExperimentalWarning)
```

# RT hit sequences assemmbled into a single files

For the purpose of reconstructing a phylogenetic tree, RT hits from all the assemblies are joind into one file

In [1]:

```
import os, sys
from Bio import SeqIO
folder = 'psiblast_ugene_RESULTS/'
dirs = os.listdir(folder)
DIRS = []
for name in dirs:
    if not '.' in name:
        DIRS.append(name)

RT_records = []


        
bad_hits = (                                 #These are crypton sequences with a short RT like hit inside their YR orf
            'Lgi:sca_27:177842:1:two_domains',
            'Lgi:sca_268:84059:1:two_domains',
            'Lgi:sca_268:84059:2:two_domains',
            'Lgi:sca_268:92085:1:two_domains',
            'Lgi:sca_268:92085:2:two_domains',
            'Lgi:sca_46:1937219:1:two_domains',
            'Lgi:sca_197:139221:1:two_domains',
            'BAS:CryptonA-1_LG:614:1:two_domains',
            'Lgi:sca_59:5984:1:two_domains',
            'Lgi:sca_225:109912:1:two_domains',
            'Lgi:sca_38:570163:1:two_domains',
            'Lgi:sca_113:551573:1:two_domains',
            'Lgi:sca_113:551191:1:two_domains',
            'Hduj:nHd.2.3.scaf00001:126730:1:two_domains', #These are elements with multiple RT sequences that cluster
            'Hduj:nHd.2.3.scaf00001:127260:1:two_domains', #with the outgroup. ie, they have adjecent LTR which wrongly 
            'Lgi:sca_75:937380:1:DIRS',                    #passed previous filters
            'Lgi:sca_18:2161112:1:two_domains',
            'Lgi:sca_268:84095:1:two_domains',
            'Nve:scaffold_182:226621:1:two_domains',
            'Ppac:Ppa_Contig83:451527:1:DIRS',
            'Acal:scaffold01513:41419:1:two_domains'
            )
for dir in DIRS:
    if os.path.exists(folder + dir + '/' + dir + '_RT_aa.fas'): 
        records = SeqIO.parse(folder + dir + '/' + dir + '_RT_aa.fas', 'fasta')
        for record in records:
            if not record.description in bad_hits:
                RT_records.append(record)


for records in [RT_records]:
    print str(len(records))
SeqIO.write(RT_records, 'RT/all_RT.fas', 'fasta')
```

```
550
```

Out[1]:

```
550
```

# YR hit sequences assemmbled into a single file

In [56]:

```
import os, sys
from Bio import SeqIO
folder = 'psiblast_ugene_RESULTS/'
dirs = os.listdir(folder)
DIRS = []
for name in dirs:
    if not '.' in name:
        DIRS.append(name)
        
YR_records = []
for dir in DIRS:
    if os.path.exists(folder + dir + '/' + dir + '_YR_aa.fas'): 
        records = SeqIO.parse(folder + dir + '/' + dir + '_YR_aa.fas', 'fasta')
        for record in records:
            YR_records.append(record)
        
SeqIO.write(YR_records, 'YR_tree/all_YR.fas', 'fasta')
```

Out[56]:

```
2753
```

# Make RT and YR files with same taxa

In [57]:

```
import os, sys
from Bio import SeqIO
folder = './'

in_YR_records = list(SeqIO.parse(folder + 'YR_tree/all_YR.fas','fasta')) 
out_YR_records = []

in_RT_records = list(SeqIO.parse(folder + 'RT/all_RT.fas','fasta')) 
out_RT_records = []


for rt_record in in_RT_records:
    for yr_record in in_YR_records:
            if rt_record.id == yr_record.id:
                out_RT_records.append(rt_record)
                out_YR_records.append(yr_record)

            
           

records =  {'YR': out_YR_records, 'RT': out_RT_records}
for i in records.keys():
    print str(len(records[i])), ' sequences selected for ', i

SeqIO.write(out_YR_records, folder + 'comparable_trees/YR.fas', 'fasta')
SeqIO.write(out_RT_records, folder + 'comparable_trees/RT.fas', 'fasta')
```

```
489  sequences selected for  RT
489  sequences selected for  YR
```

Out[57]:

```
489
```

# Make RT and YR files with same taxa and LTRs and Cryptons as outgroups respetively

In [58]:

```
from Bio import SeqIO
folder = './'


RT_no_outgroup_records = open(folder + 'comparable_trees/RT.fas', 'r').read()
YR_no_outgroup_records = list(SeqIO.parse(folder + 'comparable_trees/YR.fas', 'fasta'))

RT_with_outgroup_records = open(folder + 'comparable_trees/RT_outgrouped.fas', 'wt')
YR_with_outgroup_records = YR_no_outgroup_records


RT_outgroup = open(folder + 'LTR_sequences/RT_outgroup.fas', 'r').read()


RT_with_outgroup_records.write(RT_no_outgroup_records + RT_outgroup)
RT_with_outgroup_records.close()

all_YR_records = list(SeqIO.parse(folder + 'YR_tree/all_YR.fas','fasta'))
for yr_record in all_YR_records:
    if 'rypton' in yr_record.id:
        YR_with_outgroup_records.append(yr_record)
SeqIO.write(YR_with_outgroup_records, folder + 'comparable_trees/YR_outgrouped.fas', 'fasta')
```

Out[58]:

```
520
```

# Offensive charaters removed before alignment

The following script replaces offensive header characters and eliminates gaps in the some of the sequences that were added manually

In [59]:

```
import os, sys, re
from Bio import SeqIO


path = './'
aa_files = ['YR_tree/all_YR.fas',
            'comparable_trees/RT.fas',
            'comparable_trees/RT_outgrouped.fas',
            'comparable_trees/YR.fas',
            'comparable_trees/YR_outgrouped.fas']


for aa_file in aa_files:
    corrected_filename = aa_file.split('.')[0] + '_corrected.fas'
    corrected_file = open(path + corrected_filename,'wt')
    records = SeqIO.parse(path + aa_file, 'fasta')
    for record in records:
        record.description = re.sub('\:','_',record.description)
        record.description = re.sub('\;','_',record.description)
        record.description = re.sub('\|','_',record.description)
        record.description = re.sub('\*','_',record.description)
        record.description = re.sub('\-','_',record.description)
        record.description = re.sub('\,','_',record.description)
        record.description = re.sub('\/','_',record.description)
        record.description = re.sub(' ','_',record.description)
        record.description = re.sub('\.','_',record.description)
        corrected_file.write('>' + record.description.split(' ')[0] + '\n')
        sequence = re.sub('\*','X',str(record.seq))
        sequence = re.sub('J','X',sequence)
        sequence = re.sub('-','',sequence)
        corrected_file.write(sequence + '\n')
    corrected_file.close()
```

# YR sequences shorter than 90 bp are removed from full YR tree

In [60]:

```
from Bio import SeqIO
minimum_length = 90
path = './'
trimmed_records = []

records = list(SeqIO.parse(path + 'YR_tree/all_YR_corrected.fas', 'fasta'))

for record in records:
    res_count = 0
    sequence = str(record.seq)
    for res in sequence:
        if not res == '-':
            res_count += 1
            
    if res_count >= minimum_length:
        trimmed_records.append(record)

SeqIO.write(trimmed_records, path + 'YR_tree/all_YR_corrected.fas', 'fasta')
```

Out[60]:

```
1842
```

# Sequences aligned with mafft defaults

http://mafft.cbrc.jp/alignment/software/linux.html

In [61]:

```
import os


path = './'
aa_files = ['YR_tree/all_YR_corrected.fas',
            'comparable_trees/RT_corrected.fas',
            'comparable_trees/RT_outgrouped_corrected.fas',
            'comparable_trees/YR_corrected.fas',
            'comparable_trees/YR_outgrouped_corrected.fas']
for corrected_aa_file in aa_files:
    input_file = path + corrected_aa_file
    output_file = path + corrected_aa_file.split('.')[0] + '.mafft.fas'
    os.system('mafft ' + input_file + ' > ' + output_file)
```

# Positions with over 70% gaps are removed

This is done using the program TrimAl  
 http://trimal.cgenomics.org/

In [62]:

```
import os
from Bio import SeqIO

path = './'
aa_files = ['YR_tree/all_YR_corrected.mafft.fas',
            'comparable_trees/RT_corrected.mafft.fas',
            'comparable_trees/RT_outgrouped_corrected.mafft.fas',
            'comparable_trees/YR_corrected.mafft.fas',
            'comparable_trees/YR_outgrouped_corrected.mafft.fas']
for mafft_aa_file in aa_files:
    input_file = path + mafft_aa_file
    output_file = path + mafft_aa_file.split('.')[0] + '.mafft_trim07.fas'
    os.system('trimal -in ' + input_file + ' -out ' + output_file + ' -gt 0.3')
 
aa_files = ['YR_tree/all_YR_corrected.mafft_trim07.fas',
            'comparable_trees/RT_corrected.mafft_trim07.fas',
            'comparable_trees/RT_outgrouped_corrected.mafft_trim07.fas',
            'comparable_trees/YR_corrected.mafft_trim07.fas',
            'comparable_trees/YR_outgrouped_corrected.mafft_trim07.fas'] 

for trimal_aa_file in aa_files:
    records = list(SeqIO.parse(trimal_aa_file, 'fasta'))
    for record in records:
        record.description = ''
    SeqIO.write(records, trimal_aa_file, 'fasta')
```

# The comparable dataset are concatenated

In [63]:

```
from Bio.SeqRecord import SeqRecord
from Bio import SeqIO

path = './'
RT_records = list(SeqIO.parse(path + 'comparable_trees/RT_corrected.mafft_trim07.fas','fasta'))
YR_records = list(SeqIO.parse(path + 'comparable_trees/YR_corrected.mafft_trim07.fas', 'fasta'))
RT_YR_records = []

for rt_record in RT_records:
        for yr_record in YR_records:
            if rt_record.id == yr_record.id:
                RT_YR_record = SeqRecord(seq=rt_record.seq+yr_record.seq, id=yr_record.id)
                RT_YR_records.append(RT_YR_record)
               
SeqIO.write(RT_YR_records, path + 'comparable_trees/RTYR_corrected.mafft_trim07.fas','fasta')
```

Out[63]:

```
489
```

# YR sequences shorter than 60 bp are removed from full YR tree

In [64]:

```
from Bio import SeqIO
minimum_length = 60
path = './'
trimmed_records = []

records = list(SeqIO.parse(path + 'YR_tree/all_YR_corrected.mafft_trim07.fas', 'fasta'))

for record in records:
    res_count = 0
    sequence = str(record.seq)
    for res in sequence:
        if not res == '-':
            res_count += 1
            
    if res_count >= minimum_length:
        trimmed_records.append(record)

SeqIO.write(trimmed_records, path + 'YR_tree/all_YR_corrected.mafft_trim07_minlen30.fas', 'fasta')
```

Out[64]:

```
1662
```

# FastTree V2 reconstruction

http://www.microbesonline.org/fasttree/

In [65]:

```
import os  
path = './'
aa_files = ['YR_tree/all_YR_corrected.mafft_trim07_minlen30.fas',
            'comparable_trees/RT_corrected.mafft_trim07.fas',
            'comparable_trees/RT_outgrouped_corrected.mafft_trim07.fas',
            'comparable_trees/YR_corrected.mafft_trim07.fas',
            'comparable_trees/YR_outgrouped_corrected.mafft_trim07.fas',
            'comparable_trees/RTYR_corrected.mafft_trim07.fas'
            ]
for aa_file in aa_files:
    input_file = path + aa_file
    output_file = path + aa_file.split('.')[0] + '.tre'
    os.system('FastTreeMP -gamma -spr 4 -mlacc 2 -slownni -pseudo ' + input_file + ' > ' + output_file) #model is JTT
```

# AU test for the RT, YR and combined trees

The RT dataset excludes LTR sequences and the YR dataset excludes crypton sequences in order to have identical element representations in all three datasets. Per-site log likelihoods were computed with RAxML 7.3.5. The AU test was computed with Consel 0.20. The results did not support partition concatenation.

In [66]:

```
from ete2 import Tree
import dendropy, re, os

path = './'
if os.path.exists('consel.log'):
    os.remove('consel.log')
    
#make tree file with resolved polytomies
tree_count = 1

consel_trees = open('consel_trees.tre','wt')
for tree in ['comparable_trees/RT_corrected.tre',
             'comparable_trees/YR_corrected.tre',
             'comparable_trees/RTYR_corrected.tre'
             ]:
    os.system('echo ' + str(tree_count) + '_' + tree + ' >> consel.log')
    tree_count += 1
    t = Tree(tree)
    t.resolve_polytomy(recursive=True)
    t.set_outgroup(t&'BAS_CcNgaro3_4209_1_viper_or_Ngaro')
    consel_trees.write(t.write(format = 5) + '\n')
consel_trees.close()


for dataset in ['comparable_trees/RT_corrected.mafft_trim07.fas',
                'comparable_trees/YR_corrected.mafft_trim07.fas',
                'comparable_trees/RTYR_corrected.mafft_trim07.fas']:
   
    #convert to phylip for raxml
    d = dendropy.DataSet.get_from_path(
            dataset,
            "fasta",
    #       taxon_set=None,
            data_type="protein",
            row_type='rich') 
    d.write_to_path(
            dataset + ".phy",
            'phylip',
            taxon_set=None,
            strict=False,
            space_to_underscores=False,
            force_unique_taxon_labels=False)
    #make a correction to fasta headers
    IN = open(dataset + ".phy", 'r').readlines()
    newlines = []
    for line in IN:
        newline = re.sub(' \<unknown description\>', '', line)
        newlines.append(newline)
    OUT = open(dataset + ".phy", 'wt')
    for line in newlines:
        OUT.write(line)
    OUT.close()

    #compute per-site log likelihoods
    import os
    cwd = os.getcwd()
    dataname = dataset.split('/')[1].split('_')[0]
    files = os.listdir('./')
    for name in files:
        if 'RAxML' in name:
            os.remove(name)
    os.system('raxmlHPC -f g -s ' + dataset + '.phy -z consel_trees.tre -m PROTGAMMAJTT -n ' + dataname + ' -w ' + cwd)
    
    #au-test
    os.system('makermt -b 10 --puzzle RAxML_perSiteLLs.' + dataname)
    os.system('consel RAxML_perSiteLLs')
    os.system('echo >> consel.log; echo ' + dataset + ' >> consel.log; catpv RAxML_perSiteLLs >> consel.log' )
    

log = open('consel.log', 'r').read()
print log

files = os.listdir('./')
for name in files:
        if 'RAxML' in name:
            os.remove(name)
```

```
1_comparable_trees/RT_corrected.tre
2_comparable_trees/YR_corrected.tre
3_comparable_trees/RTYR_corrected.tre

comparable_trees/RT_corrected.mafft_trim07.fas

# reading RAxML_perSiteLLs.pv
# rank item    obs     au     np |     bp     pp     kh     sh    wkh    wsh |
#    1    1 -516.5  1.000  1.000 |  1.000  1.000  1.000  1.000  1.000  1.000 |
#    2    3  516.5  1e-39  1e-16 |      0 5e-225      0  0.073      0      0 |
#    3    2 10787.6  4e-71  8e-23 |      0      0      0      0      0      0 |


comparable_trees/YR_corrected.mafft_trim07.fas

# reading RAxML_perSiteLLs.pv
# rank item    obs     au     np |     bp     pp     kh     sh    wkh    wsh |
#    1    2 -560.7  1.000  1.000 |  1.000  1.000  1.000  1.000  1.000  1.000 |
#    2    3  560.7  3e-08  2e-09 |      0 3e-244      0      0      0      0 |
#    3    1 2840.6  5e-60  3e-20 |      0      0      0      0      0      0 |


comparable_trees/RTYR_corrected.mafft_trim07.fas

# reading RAxML_perSiteLLs.pv
# rank item    obs     au     np |     bp     pp     kh     sh    wkh    wsh |
#    1    3 -1685.2  1.000  1.000 |  1.000  1.000  1.000  1.000  1.000  1.000 |
#    2    1 1685.2  2e-06  2e-07 |      0      0      0  0.050      0      0 |
#    3    2 9395.1  6e-65  1e-20 |      0      0      0      0      0      0 |
```

# Node name replacement after preliminary review

Following a preliminary tree reconstruction, the diagrams of elements that seemed misplaced were examined and their classification was manually corrected if the pipeline indication did not seem to feet the diagram (see above for reasons why).

In [67]:

```
from ete2 import Tree, NodeStyle, TreeStyle, TextFace, PhyloTree
import sys

path = './'
tre_files = ['YR_tree/all_YR_corrected.tre',
            'comparable_trees/RT_corrected.tre',
            'comparable_trees/RT_outgrouped_corrected.tre',
            'comparable_trees/YR_corrected.tre',
            'comparable_trees/YR_outgrouped_corrected.tre',
            'comparable_trees/RTYR_corrected.tre'
            ]
name_replacements = open(path + 'renamed_nodes.txt','r').readlines()

for tre_file in tre_files:
    replaced_nodes = open(path + tre_file.split('.')[0] + '_replaced_nodes.txt', 'wt')
    replaced_count = 0
    tree = Tree(path + tre_file)
    for line in name_replacements:
        line = line.rstrip('\n')
        (old_name, new_name) = line.split('\t')
        if old_name in tree.get_leaf_names():
            (tree&old_name).name = new_name
            replaced_count += 1
            replaced_nodes.write(old_name + '\t' + new_name + '\n')
    print 'Replaced ' + str(replaced_count) + ' node names out of ' + str(len(tree)) + ' in tree ' + tre_file
    print 'Replacements are in ' + tre_file.split('.')[0] + '_replaced_nodes.txt'
    print 
    replaced_nodes.write('Replaced ' + str(replaced_count) + ' node names out of ' + str(len(tree)) + ' in tree ' + tre_file + 
                         '\nReplacements are in ' + tre_file.split('.')[0] + '_replaced_nodes.txt')
    replaced_nodes.close()
    tree.write(outfile= path + tre_file.split('.')[0] + '_reviewed.tre', format=1, features=['support'])
```

```
Replaced 9 node names out of 1662 in tree YR_tree/all_YR_corrected.tre
Replacements are in YR_tree/all_YR_corrected_replaced_nodes.txt

Replaced 1 node names out of 489 in tree comparable_trees/RT_corrected.tre
Replacements are in comparable_trees/RT_corrected_replaced_nodes.txt

Replaced 1 node names out of 520 in tree comparable_trees/RT_outgrouped_corrected.tre
Replacements are in comparable_trees/RT_outgrouped_corrected_replaced_nodes.txt

Replaced 1 node names out of 489 in tree comparable_trees/YR_corrected.tre
Replacements are in comparable_trees/YR_corrected_replaced_nodes.txt

Replaced 1 node names out of 520 in tree comparable_trees/YR_outgrouped_corrected.tre
Replacements are in comparable_trees/YR_outgrouped_corrected_replaced_nodes.txt

Replaced 1 node names out of 489 in tree comparable_trees/RTYR_corrected.tre
Replacements are in comparable_trees/RTYR_corrected_replaced_nodes.txt
```

# Automatic node collapse

Nodes were collapsed if they had a sh-like support grater than 0.7 in the reduced YR tree or 0.9 in the full YR tree and if they did not contain conflicting classificaionts. In addition, in unsupported clades (sh-support < 0.95) nodes that have long branches (4 times the median branch length in the clade) are removed for the sake of the comparisson between the full YR tree and the reduced YR tree.

In [1]:

```
from ete2 import Tree, NodeStyle, TreeStyle, TextFace, PhyloTree, CircleFace, PieChartFace
import sys, numpy
path = './'

def blen_cutoff(node,brlns_cutoff_factor):
    blens = []
    for br in node.traverse():
        if not br.name == node.name and not br.dist == 0:
            blens.append(float(br.dist))
    return numpy.median(blens)*brlns_cutoff_factor


tree_string_files = [
                     'YR_tree/all_YR_corrected_reviewed.tre',
                     #'comparable_trees/RT_corrected_reviewed.tre',
                     'comparable_trees/RT_outgrouped_corrected_reviewed.tre',
                     #'comparable_trees/RTYR_corrected_reviewed.tre',
                     #'comparable_trees/YR_corrected_reviewed.tre',
                     'comparable_trees/YR_outgrouped_corrected_reviewed.tre'
                    ]
   


for tree_string_file in tree_string_files:
    support_cutoff = 0.01
    if tree_string_file ==  'YR_tree/all_YR_corrected_reviewed.tre':
        support_cutoff = 0.9 #higher cutoff for phylo-placing than for just phylo presenting
    elif tree_string_file ==  'comparable_trees/YR_outgrouped_corrected_reviewed.tre':
        support_cutoff = 0.7 #higher cutoff for phylo-placing than for just phylo presenting
    
        
    tree_string = open(path + tree_string_file, 'r').read()
    t = Tree(tree_string, format=1)
    print len(t)
    node_number = 1 #We are going to give running numbers to all the nodes for easier access later on
    
    for n in t.traverse():
        if n.is_leaf and 'rypton' in n.name:
            n.name = n.name + '_crypton' # This will make the annotation easier
        if n.support < support_cutoff:
            n.delete()                   # multifrucate unsupported bifrucarions
            
    print_collapsed_nodes = 1            # will make a table of the collaped nodes, named by running numbers, and their 
                                         # leaf content
    
    for n in t.traverse():
        if not n.is_leaf():
            organisms_in_node = {       # will be used to count taxa in collapsed nodes     
                    'PAT': 0,
                    'BAS': 0,
                    'RET': 0,
                    'Mflo': 0,
                    'Dviv': 0,
                    'Ebre': 0,
                    'Rcul': 0,
                    'Avit': 0,
                    'Asuu': 0,
                    'Bxyl': 0,
                    'Hbac': 0,
                    'Haor': 0,
                    'Hcon': 0,
                    'Srat': 0,
                    'briC': 0,
                    'Cele': 0,
                    'Cjap': 0,
                    'Bmal': 0,
                    'Cbre': 0,
                    'Crem': 0,
                    'Gpal': 0,
                    'Lsig': 0,
                    'Ppac': 0,
                    'Lloa': 0,
                    'Mhap': 0,
                    'Minc': 0,
                    'C5sp': 0,
                    'Dimm': 0,
                    'Ooch': 0,
                    'Ovol': 0,
                    'Otip': 0,
                    'Pred': 0,
                    'C11sp': 0,
                    'Cjap': 0,
                    'Cang': 0,
                    'Tmur': 0,
                    'Tspi': 0,
                    'Wban': 0,
                    'Acas': 0,
                    'Agam': 0,
                    'Acal': 0,
                    'Dpul': 0,
                    'Dsim': 0,
                    'Lgi': 0,
                    'Nvi': 0,
                    'Nve': 0,
                    'Ath': 0,
                    'Vcar': 0,
                    'Alyr': 0,
                    'Hduj': 0
                    }
            elements_in_node = {     #will be used to count element types in collapsed nodes
                    'dirs1_like': 0,
                    'pat1_like': 0,
                    'PAT': 0,
                    'viper_or_Ngaro': 0,
                    'kangaroo': 0,
                    'DIRS': 0,
                    'domains_inverted': 0,
                    'two_domains': 0,
                    'YR_only': 0,
                    'crypton': 0,
                    'DERIVED': 0,    
                    'GYPSY': 0,
                    'COPIA': 0,
                    'BEL': 0
                    }
            # node will be collapsed only if all the leafs can be placed in only one of these arrays
            dirs1_hirarchy = ('dirs1_like', 'DIRS', 'two_domains', 'YR_only','DERIVED')
            pat_like_hirarchy = ('pat1_like', 'DIRS', 'two_domains', 'YR_only','DERIVED')
            toc_hirarchy = ('PAT', 'DIRS', 'two_domains', 'YR_only','DERIVED')
            viper_or_Ngaro_hirarchy = ('viper_or_Ngaro', 'two_domains', 'YR_only','DERIVED')
            kangaroo_hirarchy = ('kangaroo', 'domains_inverted','two_domains', 'YR_only','DERIVED', 'DIRS')
            DIRS_hirarchy = ('DIRS', 'two_domains', 'YR_only','DERIVED')
            domains_inverted_hirarchy = ('domains_inverted','two_domains', 'YR_only','DERIVED')
            two_domains_hirarchy = ('two_domains','domains_inverted', 'YR_only','DERIVED')
            crypton_hirarchy = ('crypton', 'YR_only')
            YR_hirarchy = crypton_hirarchy 
            GYPSY_hirarchy = ('GYPSY')
            COPIA_hirarchy = ('COPIA')
            BEL_hirarchy = ('BEL')
        
        
            n.name = 'node' + str(node_number) #naming the node
            node_number += 1
        
            # prepare species counts and element counts to be added to all nodes
            for leaf in n:
                    for orgn in organisms_in_node.keys(): # orgn is the species key
                        search_length = len(orgn)
                        if leaf.name[:search_length] == orgn: # if begining of the leaf name same as orgn
                            organisms_in_node[orgn] += 1      # count it
                    for element in elements_in_node.keys():   
                        search_length = len(element)                    
                        if leaf.name[-1*search_length:] == element:  # if end of the leaf name same as element
                            elements_in_node[element] += 1           # count it 
            for orgn in organisms_in_node.keys(): # get read of species codes with zero count 
                if organisms_in_node[orgn] == 0:
                    organisms_in_node.pop(orgn, None) 
            for element in elements_in_node.keys(): # get read of elements with zero count
                if elements_in_node[element] == 0:
                    elements_in_node.pop(element, None)
            n.add_features(species=organisms_in_node, elements=elements_in_node) # add speceis count and element counts
                                                                                 # as node features
    # write tree with node names for future reference        
    t.write(outfile=path + tree_string_file.split('.')[0] + '_internal_nodes.tre', format=1, features=["support"])
    collapsed_nodes_file = open(path + tree_string_file.split('.')[0] + '_collapsed_nodes.txt','wt')
    collapsed_nodes = []
    

    # decside which node to collapse based on the presence absence of conflicting element forms
    for n in t.traverse():
        if not n.is_leaf():
            collapse = 1

            if 'dirs1_like' in n.elements.keys():
                for name in n.elements.keys():
                    if not name in dirs1_hirarchy:
                        collapse = 0
            elif 'pat1_like' in n.elements.keys():
                for name in n.elements.keys():
                    if not name in pat_like_hirarchy:
                        collapse = 0 
            elif 'PAT' in n.elements.keys():
                for name in n.elements.keys():
                    if not name in toc_hirarchy:
                        collapse = 0
            elif 'kangaroo' in n.elements.keys():
                for name in n.elements.keys():
                    if not name in kangaroo_hirarchy:
                        collapse = 0
            elif 'viper_or_Ngaro' in n.elements.keys():
                for name in n.elements.keys():
                    if not name in viper_or_Ngaro_hirarchy:
                        collapse = 0
            elif 'DIRS' in n.elements.keys():
                for name in n.elements.keys():
                    if not name in DIRS_hirarchy:
                        collapse = 0
            elif 'domains_inverted' in n.elements.keys():
                for name in n.elements.keys():
                    if not name in domains_inverted_hirarchy:
                        collapse = 0
            elif 'two_domains' in n.elements.keys():
                for name in n.elements.keys():
                    if not name in two_domains_hirarchy:
                        collapse = 0
            elif 'COPIA' in n.elements.keys():
                for name in n.elements.keys():
                    if not name in COPIA_hirarchy:
                        collapse = 0
            elif 'BEL' in n.elements.keys():
                for name in n.elements.keys():
                    if not name in BEL_hirarchy:
                        collapse = 0
            elif 'GYPSY' in n.elements.keys():
                for name in n.elements.keys():
                    if not name in GYPSY_hirarchy:
                        collapse = 0
            elif 'crypton' in n.elements.keys():
                for name in n.elements.keys():
                    if not name in crypton_hirarchy:
                        collapse = 0
            elif 'YR_only' in n.elements.keys():
                for name in n.elements.keys():
                    if not name in YR_hirarchy:
                        collapse = 0
            if collapse == 1:
                if tree_string_file == 'YR_tree/all_YR_corrected_reviewed.tre' and n.support < 0.95:
                    n_descendants = n.get_descendants()
                    cutoff = blen_cutoff(n,4)
                    for d in n_descendants:
                         if d.dist > cutoff:
                             d.detach()
                if print_collapsed_nodes == 1: #print all leaves in a file. It will be used later 
                    for leaf in n:
                        if not 'node' in leaf.name:
                            collapsed_nodes.append(leaf.name + '\t' + n.name + '\t')
                for C in n.get_descendants(): # and remove their leaves
                        C.detach() 
        
    
    
    
    ts = TreeStyle()
    ts.show_leaf_name = False
    #ts.scale = 150
    ts.show_branch_support = False
    t.ladderize(1)
    R = t.get_midpoint_outgroup()

    ns = NodeStyle()
    ns['hz_line_width'] = 5
    ns['vt_line_width'] = 5
    ns['fgcolor'] = 'black'
    ns['size'] = 0
    
    
    t.set_outgroup(R)

    for node in t.traverse(): 
        node.set_style(ns)
        if node.is_leaf():           
            if not 'node' in node.name:    #add faces to leaves
                color = 'gray'
                if 'rypton' in node.name:
                    color = 'sandybrown'
                    collapsed_nodes_file.write(node.name + '\t' + node.name + '\t' + 'Crypton' + '\n')
                elif 'kangaroo' in node.name:
                    color = 'red'
                    collapsed_nodes_file.write(node.name + '\t' + node.name + '\t' + 'kangaroo' + '\n')
                elif 'dirs1' in node.name:
                    color = 'darkviolet'
                elif 'PAT' in node.name:
                    color = 'green'
                elif 'Ngaro' in node.name:
                    color = 'black'
                    if ('BAS' in node.name or 'RET' in node.name) and not 'DIRS' in node.name:
                        collapsed_nodes_file.write(node.name + '\t' + node.name + '\t' + 'Ngaro' + '\n')
                elif 'ypsy' in node.name:
                    node.name = 'GYPSY'
                    color = 'black'
                name_parts = node.name.split('_')
                name = name_parts[0]
                get_name_part = 0
                for part in name_parts:
                    if get_name_part == 1:
                        name = name + '_' + part
                    elif part == '1':
                        get_name_part = 1
                hola = TextFace(name, fsize=15, fgcolor=color)    
                #node.add_face(hola, column=0, position = "branch-right")
                if node.name == 'GYPSY':
                    node.add_face(hola, column=0, position = "branch-right")
                elif color == 'gray':
                    node.add_face(CircleFace(radius = 10, color = 'gray', style = 'sphere'), column=0, position = "branch-right")
                else:
                    node.add_face(CircleFace(radius = 10, color = color), column=0, position = "branch-right")
                
                
                if 'RET_' in node.name or 'PAT_' in node.name or 'BAS_' in node.name:
                    if 'dirs1_like' in node.name:
                        collapsed_nodes_file.write(node.name + '\t' + node.name + '\t' + 'dirs1_like' + '\n')
                    elif 'pat1_like' in node.name:
                        collapsed_nodes_file.write(node.name + '\t' + node.name + '\t' + 'pat1_like' + '\n')
                    elif '_PAT' in node.name:
                        collapsed_nodes_file.write(node.name + '\t' + node.name + '\t' + 'PAT' + '\n')
                    else:
                        collapsed_nodes_file.write(node.name + '\t' + node.name + '\t' + 'None' + '\n')
            elif 'node' in node.name:  # add faces to collapsed nodes
                bcolor = 'white'
                species = ''
                count = 0
                for key in node.species.keys():
                    species =species + key + '_' + str(node.species[key]) + '_'
                    count += node.species[key]
                elements = ''
                for key in node.elements.keys():
                    elements = elements + key + '_' + str(node.elements[key]) + ' '
                verdict = 'None'
                verdict_color = 'black'
                if 'dirs1_like' in elements:
                    verdict = 'dirs1_like'
                    verdict_color = 'darkviolet'
                elif 'pat1_like' in elements:
                    verdict = 'pat1_like'
                    verdict_color = 'blue'
                elif 'PAT' in elements:
                    verdict = 'PAT'
                    verdict_color = 'green'
                elif 'kangaroo' in elements:
                    verdict = 'kangaroo'
                    verdict_color = 'red'
                elif 'Ngaro' in elements:
                    verdict = 'Ngaro'
                    verdict_color = 'black'
                elif 'crypton' in elements:
                    verdict = 'Crypton'
                    verdict_color = 'sandybrown'
                elif 'BEL' in elements:
                    verdict = 'BEL'
                    verdict_color = 'black'
                elif 'COPIA' in elements:
                    verdict = 'COPIA'
                    verdict_color = 'black'
                elif 'GYPSY' in elements:
                    verdict = 'GYPSY'
                    verdict_color = 'black'
                for line in collapsed_nodes:
                    if line.split('\t')[1] == node.name:
                        collapsed_nodes_file.write(line + verdict + '\n')
                el = TextFace(elements, fsize=10, fgcolor = 'dimgray')
                if bcolor == 'black':
                    el.fgcolor = 'white'
                sp = TextFace(species, fsize=10, fgcolor = 'dimgray')
                if count < 15:
                    count = 15
                #if len(species) > 0:
                    #species_line_to_pie_chart_face(node, species,species_groupings,float(count)*1+5)
                #sp.border.width = 1
                
                vr = TextFace(verdict, fsize=count, fgcolor=verdict_color)
                #if verdict == 'pat1_like':
                    #vr.background.color = 'lightgray'
                #node.add_face(sp, column=2, position = "branch-right")
                
                #node.add_face(el, column=2, position = "branch-right")
                if not verdict == 'None' and not verdict == 'COPIA' and not verdict == 'BEL' and not verdict == 'GYPSY':
                    node.add_face(CircleFace(radius = 10, color = verdict_color), column=0, position = "branch-right")
                    #node.add_face(vr, column=1, position = "branch-right")
                   
                elif not verdict == 'COPIA' and not verdict == 'BEL' and not verdict == 'GYPSY':
                    node.add_face(CircleFace(radius = 10, color = 'gray', style = 'sphere'), column=0, position = "branch-right")
                else:
                    node.add_face(vr, column=1, position = "branch-right")
                hola = TextFace(node.name, fsize=10, fgcolor='gray')    
                #node.add_face(hola, column=0, position = "branch-right")
                score = TextFace(str(node.support),fsize=6, fgcolor='black')
                #node.add_face(score,column=0, position = "branch-right")
                if node.support == 1:
                    node.add_face(CircleFace(radius = 5, color = 'black'),column=0, position = "float")
                elif node.support < 1 and node.support >= 0.95:
                    node.add_face(CircleFace(radius = 5, color = 'dimgray'),column=0, position = "float")
                elif node.support < 0.95 and node.support >= 0.9:
                    node.add_face(CircleFace(radius = 5, color = 'gray'),column=0, position = "float")
                elif node.support < 0.9 and node.support >= 0.8:
                    node.add_face(CircleFace(radius = 5, color = 'gainsboro'),column=0, position = "float")
        else: #add faces to internal nodes
            hola = TextFace(node.name, fsize=10, fgcolor='gray')    
            #node.add_face(hola, column=0, position = "branch-right")
            if node.support == 1:
                    node.add_face(CircleFace(radius = 5, color = 'black'),column=0, position = "float")
            elif node.support < 1 and node.support >= 0.95:
                    node.add_face(CircleFace(radius = 5, color = 'dimgray'),column=0, position = "float")
            elif node.support < 0.95 and node.support >= 0.9:
                    node.add_face(CircleFace(radius = 5, color = 'gray'),column=0, position = "float")
            elif node.support < 0.9 and node.support >= 0.8:
                    node.add_face(CircleFace(radius = 5, color = 'gainsboro'),column=0, position = "float")
            
    
    t.dist = 0
    t.support = 0
    t.show(tree_style = ts)
    #t.render(path + tree_string_file.split('.')[0] + '_Figure.tif', w = 1000, tree_style = ts)
    collapsed_nodes_file.close()
```

```
1662
520
520
```

# Assign clade from the YR tree to clade in the reduced\_YR tree

**Note: If the trees are recalculated the output of this cell will change**.

In [2]:

```
reduced_tree_nodes = open('comparable_trees/YR_outgrouped_corrected_reviewed_collapsed_nodes.txt').readlines()
full_tree_nodes = open('YR_tree/all_YR_corrected_reviewed_collapsed_nodes.txt').readlines()

node_to_nodes = {}


for line in reduced_tree_nodes:
    leaf_name, node_name, verdict = line.rstrip('\n').split('\t')
    if not verdict == 'None' and not node_name in node_to_nodes.keys():
        node_to_nodes[node_name] = {}
        node_to_nodes[node_name]['verdict'] = verdict
        node_to_nodes[node_name]['nodes'] = []
        
                
for full_t_line in full_tree_nodes:
    full_t_leaf_name, full_t_node_name, full_t_verdict = full_t_line.rstrip('\n').split('\t')
    for line in reduced_tree_nodes:
        leaf_name, node_name, verdict = line.rstrip('\n').split('\t')
        if full_t_leaf_name == leaf_name:
            if (full_t_verdict == 'None' or
                full_t_verdict == verdict
                ):
                if (node_name in node_to_nodes.keys() and 
                    not full_t_node_name in node_to_nodes[node_name]['nodes']):
                    node_to_nodes[node_name]['nodes'].append(full_t_node_name)

dict_for_figure = {}
for node in node_to_nodes.keys():
    node_verdict = node_to_nodes[node]['verdict']
    if node_verdict in ['dirs1_like','pat1_like','kangaroo']:
        if not node_verdict in dict_for_figure.keys():
            dict_for_figure[node_verdict] = {}
            dict_for_figure[node_verdict]['reduced'] = []
            dict_for_figure[node_verdict]['full'] = []
        dict_for_figure[node_verdict]['reduced'].append(node)
        for n in node_to_nodes[node]['nodes']:
            if not n in dict_for_figure[node_verdict]['full']:
                dict_for_figure[node_verdict]['full'].append(n)
    else:
        key = node_verdict + '__' + node
        dict_for_figure[key] = {}
        dict_for_figure[key]['reduced'] = [node]
        dict_for_figure[key]['full'] = node_to_nodes[node]['nodes']
        
print dict_for_figure
```

```
{'PAT__node235': {'full': ['node78'], 'reduced': ['node235']}, 'PAT__node236': {'full': [], 'reduced': ['node236']}, 'PAT__node237': {'full': ['node210'], 'reduced': ['node237']}, 'Crypton__node165': {'full': ['RET_CryptonCn1_1358_1_YR_only_crypton', 'node148'], 'reduced': ['node165']}, 'Ngaro__node167': {'full': ['node102', 'node82', 'BAS_DIRS_53_XT_3142_1_viper_or_Ngaro', 'node263', 'node448', 'node546'], 'reduced': ['node167']}, 'Crypton__BAS_CryptonA_1_OL_300_1_YR_only_crypton': {'full': ['BAS_CryptonA_1_OL_300_1_YR_only_crypton'], 'reduced': ['BAS_CryptonA_1_OL_300_1_YR_only_crypton']}, 'Ngaro__node222': {'full': [], 'reduced': ['node222']}, 'pat1_like': {'full': ['node224', 'node219', 'node119', 'node217'], 'reduced': ['node194', 'node197', 'node196', 'node262', 'node260', 'node325']}, 'Crypton__BAS_CryptonF_6_PI_451_1_YR_only_crypton': {'full': ['node204'], 'reduced': ['BAS_CryptonF_6_PI_451_1_YR_only_crypton']}, 'Crypton__BAS_CryptonV_1_NV_2095_1_YR_only_crypton': {'full': ['BAS_CryptonV_1_NV_2095_1_YR_only_crypton'], 'reduced': ['BAS_CryptonV_1_NV_2095_1_YR_only_crypton']}, 'PAT__node266': {'full': ['node133'], 'reduced': ['node266']}, 'PAT__node229': {'full': ['node213'], 'reduced': ['node229']}, 'Crypton__node173': {'full': ['BAS_Crypton_10_CGi_839_1_YR_only_crypton', 'BAS_Crypton_9_CGi_1469_1_YR_only_crypton', 'BAS_CryptonA_3_SK_54_1_YR_only_crypton', 'node240'], 'reduced': ['node173']}, 'PAT__node160': {'full': ['node79', 'node56'], 'reduced': ['node160']}, 'kangaroo': {'full': ['node93'], 'reduced': ['Vcar_scaffold_38_112287_1_kangaroo']}, 'Ngaro__node135': {'full': ['node102', 'node155'], 'reduced': ['node135']}, 'dirs1_like': {'full': ['node104', 'node103', 'node382', 'node98', 'node403', 'node299', 'node298', 'node293', 'node78', 'node112', 'node294', 'node381', 'node287', 'node111', 'node110', 'node109', 'node95', 'node120', 'node105', 'RET_RoDirs1_3186_1_dirs1_like', 'node373', 'BAS_DIRS_4_SK_3309_1_DIRS', 'node295', 'node107', 'node291', 'node290', 'node379', 'node376', 'node377', 'node292'], 'reduced': ['node77', 'node73', 'node17', 'node32', 'node30', 'node191', 'node193', 'node8', 'node95', 'node94', 'node50', 'node131', 'node180', 'RET_TcDirs1_3616_1_dirs1_like', 'RET_RoDirs1_3186_1_dirs1_like', 'node68', 'node46', 'node51', 'node3', 'node66', 'node161', 'node121', 'node122', 'node5', 'node4', 'node7']}, 'Crypton__BAS_Crypton_1_NV_637_1_YR_only_crypton': {'full': ['node683'], 'reduced': ['BAS_Crypton_1_NV_637_1_YR_only_crypton']}, 'PAT__node192': {'full': ['node90'], 'reduced': ['node192']}, 'Crypton__BAS_CryptonA_1_LG_614_1_two_domains_crypton': {'full': ['BAS_CryptonA_1_LG_614_1_two_domains_crypton'], 'reduced': ['BAS_CryptonA_1_LG_614_1_two_domains_crypton']}, 'Crypton__node134': {'full': ['node215'], 'reduced': ['node134']}, 'Crypton__BAS_CryptonS_2_PU_272_1_YR_only_crypton': {'full': ['BAS_CryptonS_2_PU_272_1_YR_only_crypton'], 'reduced': ['BAS_CryptonS_2_PU_272_1_YR_only_crypton']}, 'Crypton__BAS_Crypton_1_NV_309_1_YR_only_crypton': {'full': [], 'reduced': ['BAS_Crypton_1_NV_309_1_YR_only_crypton']}}
```

## Find PAT clades that have sequences with zinc finger and rename pat1-like

**elements with ZF**

In [3]:

```
TEs_with_ZF = """Lgi_150_453169_PAT 1 pat1_Ngaro
Lgi_59_1690142_DIRS 1 pat1_Ngaro
Lgi_59_1685024_DIRS 1 pat1_Ngaro
Lgi_246_5989_DIRS 1 pat1_Ngaro
Lgi_246_5423_DIRS 1 pat1_Ngaro
Nve_318_58153_PAT 1 pat1_Ngaro
Nve_318_58227_PAT 1 pat1_Ngaro
Hcon_0059030_17723_pat1-like 1 pat1_Ngaro
Hcon_0059030_17196_pat1-like 1 pat1_Ngaro
Rcul_11614_5929_DIRS 1 pat1_Ngaro
Rcul_65775_27385_DIRS 1 pat1_Ngaro"""

TEs_with_ZF = TEs_with_ZF.split('\n')
collapsed_nodes = open('comparable_trees/YR_outgrouped_corrected_reviewed_collapsed_nodes.txt', 'r').readlines()

keys_to_replace_with = []

for line in collapsed_nodes:
    line_contig_and_start = line.split('_')[2] + '_' + line.split('_')[3]
    node_number = line.split('\t')[1]
    for hit in TEs_with_ZF:
        hit_contig_and_start = hit.split('_')[1] + '_' + hit.split('_')[2]
        hit_verdict = hit.split('_')[3]
        hit_verdict = hit_verdict.split(' ')[0]
        if hit_contig_and_start == line_contig_and_start and hit_verdict == 'PAT':
            print hit_contig_and_start, line_contig_and_start
            for key in dict_for_figure.keys():
                if [node_number] == dict_for_figure[key]['reduced']:
                    print key
                    node_in_key = key.split('__')[1]
                    new_key = 'pat1_like' + '__' + node_number
                    keys_to_replace_with.append([key, new_key])
                    
for replace in keys_to_replace_with:
    if replace[0] in dict_for_figure.keys():
       dict_for_figure[replace[1]] = dict_for_figure[replace[0]]
       del dict_for_figure[replace[0]]
```

```
318_58153 318_58153
PAT__node235
150_453169 150_453169
PAT__node160
318_58227 318_58227
PAT__node160
```

# Echinoderm for Pat1-like related

**This is a list of leaves form the nodes 235 and 160 in the small YR tree  
and the leaves from associated nodes in the full YR tree** Vcar\_scaffold\_32\_252119\_1\_DIRS  
Vcar\_scaffold\_4\_1088875\_1\_YR\_only  
Vcar\_scaffold\_9\_651715\_1\_YR\_only  
Vcar\_scaffold\_84\_98418\_1\_YR\_only  
Vcar\_scaffold\_27\_1811040\_1\_YR\_only  
Vcar\_scaffold\_1\_1672594\_1\_two\_domains  
Vcar\_scaffold\_31\_1028164\_1\_DIRS  
Vcar\_scaffold\_9\_264930\_1\_PAT  
Vcar\_scaffold\_2\_3631086\_1\_PAT  
Vcar\_scaffold\_1\_3941792\_1\_PAT  
Vcar\_scaffold\_21\_2126231\_1\_PAT  
Vcar\_scaffold\_21\_80243\_1\_PAT  
Vcar\_scaffold\_9\_3478209\_1\_PAT  
Vcar\_scaffold\_2\_1384943\_1\_PAT  
Vcar\_scaffold\_684\_1328\_1\_two\_domains  
Vcar\_scaffold\_21\_4474\_1\_PAT  
Vcar\_scaffold\_77\_345705\_1\_YR\_only  
BAS\_DIRS\_3\_CGi\_3386\_1\_DIRS  
Lgi\_sca\_38\_1319572\_1\_two\_domains  
Lgi\_sca\_13\_1596769\_1\_DIRS  
Lgi\_sca\_246\_5423\_1\_DIRS  
Lgi\_sca\_114\_28036\_1\_two\_domains  
Lgi\_sca\_31\_2840409\_1\_DIRS  
Lgi\_sca\_59\_1685024\_1\_DIRS  
BAS\_DIRS\_1\_RO\_3043\_1\_DIRS  
Lgi\_sca\_150\_453169\_1\_PAT  
**RET\_SpPat1\_3157\_1\_DIRS**  
Rcul\_scaffold5760\_1\_size11614\_5929\_1\_DIRS  
Nve\_scaffold\_318\_58153\_1\_PAT  
Nve\_scaffold\_17\_1547274\_1\_DIRS  
Nve\_scaffold\_318\_58227\_1\_PAT

# Collapse groups for figure 3

The groups in dict\_for\_figure are collapsed into bins according to their feature order based calssification. The phylogenetic information is retained in figure 2.

In [4]:

```
collapse_dict_for_figure = {}
bins = ['Ngaro','PAT','kangaroo','crypton','pat1_like','dirs1_like']
for bin in bins:
    collapse_dict_for_figure[bin] = {'full': [],
                                     'reduced': []}
    for key in dict_for_figure.keys():
        if bin in key:
            collapse_dict_for_figure[bin]['full'] = collapse_dict_for_figure[bin]['full'] + dict_for_figure[key]['full']
            collapse_dict_for_figure[bin]['reduced'] = collapse_dict_for_figure[bin]['reduced'] + dict_for_figure[key]['reduced']
print collapse_dict_for_figure
```

```
{'PAT': {'full': ['node210', 'node133', 'node213', 'node90'], 'reduced': ['node236', 'node237', 'node266', 'node229', 'node192']}, 'crypton': {'full': ['BAS_CryptonA_1_OL_300_1_YR_only_crypton', 'node204', 'BAS_CryptonV_1_NV_2095_1_YR_only_crypton', 'node683', 'BAS_CryptonA_1_LG_614_1_two_domains_crypton', 'BAS_CryptonS_2_PU_272_1_YR_only_crypton'], 'reduced': ['BAS_CryptonA_1_OL_300_1_YR_only_crypton', 'BAS_CryptonF_6_PI_451_1_YR_only_crypton', 'BAS_CryptonV_1_NV_2095_1_YR_only_crypton', 'BAS_Crypton_1_NV_637_1_YR_only_crypton', 'BAS_CryptonA_1_LG_614_1_two_domains_crypton', 'BAS_CryptonS_2_PU_272_1_YR_only_crypton', 'BAS_Crypton_1_NV_309_1_YR_only_crypton']}, 'Ngaro': {'full': ['node102', 'node82', 'BAS_DIRS_53_XT_3142_1_viper_or_Ngaro', 'node263', 'node448', 'node546', 'node102', 'node155'], 'reduced': ['node167', 'node222', 'node135']}, 'dirs1_like': {'full': ['node104', 'node103', 'node382', 'node98', 'node403', 'node299', 'node298', 'node293', 'node78', 'node112', 'node294', 'node381', 'node287', 'node111', 'node110', 'node109', 'node95', 'node120', 'node105', 'RET_RoDirs1_3186_1_dirs1_like', 'node373', 'BAS_DIRS_4_SK_3309_1_DIRS', 'node295', 'node107', 'node291', 'node290', 'node379', 'node376', 'node377', 'node292'], 'reduced': ['node77', 'node73', 'node17', 'node32', 'node30', 'node191', 'node193', 'node8', 'node95', 'node94', 'node50', 'node131', 'node180', 'RET_TcDirs1_3616_1_dirs1_like', 'RET_RoDirs1_3186_1_dirs1_like', 'node68', 'node46', 'node51', 'node3', 'node66', 'node161', 'node121', 'node122', 'node5', 'node4', 'node7']}, 'pat1_like': {'full': ['node224', 'node219', 'node119', 'node217', 'node79', 'node56', 'node78'], 'reduced': ['node194', 'node197', 'node196', 'node262', 'node260', 'node325', 'node160', 'node235']}, 'kangaroo': {'full': ['node93'], 'reduced': ['Vcar_scaffold_38_112287_1_kangaroo']}}
```

# Write up table of element counts per sepcies

In [5]:

```
from ete2 import Tree, NodeStyle, TreeStyle, TextFace, PhyloTree
import numpy

reduced_tree = Tree('comparable_trees/YR_outgrouped_corrected_reviewed_internal_nodes.tre', format=1)
full_tree = Tree('YR_tree/all_YR_corrected_reviewed_internal_nodes.tre', format=1)


def get_all_leaves(tree, node_name):
    if 'node' in node_name:
        leaves = (tree&node_name).get_leaf_names()
        for leaf_name in leaves:
            if 'node' in leaf_name:
                leaves.pop(leaves.index(leaf_name))
        return leaves
    else:
        print 'trying to get leaves from a leaf'


def merge_objects(a, b):
    merged = []
    if isinstance(a, list) and isinstance(b, list):
        merged = a
        for i in b:
            if not i in merged:
                merged.append(i)
    elif isinstance(a, list) and not isinstance(b, list):
       merged = a
       if not b in merged:
            merged.append(b)
    elif not isinstance(a, list) and isinstance(b, list):
        merged = b
        if not a in merged:
            merged.append(a)
    elif not isinstance(a, list) and not isinstance(b, list):
        if not a == b:
            merged = (a, b)
    return merged


count_elements_per_species = {}

all_leaf_names_per_element = {}


all_elements_nodes = collapse_dict_for_figure

for element in all_elements_nodes.keys():  #compose list of all nodes
    full_list_of_leaves_for_this_elements = []
    for tree in all_elements_nodes[element].keys():
        t = reduced_tree
        if tree == 'full':
            t = full_tree
        if isinstance(all_elements_nodes[element][tree], str):
            if not 'node' in all_elements_nodes[element][tree]:
                full_list_of_leaves_for_this_elements = merge_objects(full_list_of_leaves_for_this_elements, all_elements_nodes[element][tree])
            elif 'node' in all_elements_nodes[element][tree]:
                leaves = get_all_leaves(t, all_elements_nodes[element][tree])
                full_list_of_leaves_for_this_elements = merge_objects(full_list_of_leaves_for_this_elements, leaves)
        else:
            for node_name in all_elements_nodes[element][tree]:
                if not 'node' in node_name:
                    full_list_of_leaves_for_this_elements = merge_objects(full_list_of_leaves_for_this_elements, node_name)
                elif 'node' in node_name:
                    leaves = get_all_leaves(t, node_name)
                    full_list_of_leaves_for_this_elements = merge_objects(full_list_of_leaves_for_this_elements, leaves)
    all_leaf_names_per_element[element] = full_list_of_leaves_for_this_elements

    
#Write a file of all the elements that were classified phylogenetically
#Write a file of all the elements from REFERECE sequences that were classified phylogenetically

elements_list = open('./psiblast_ugene_RESULTS/all_elements_found.txt','wt')
DB_elements_list = open('./psiblast_ugene_RESULTS/all_DB_elements_found.txt','wt') 
for element in  all_leaf_names_per_element.keys():
    elements_list.write(element + '\n-------\n')
    DB_elements_list.write(element + '\n-------\n')
    for leaf in all_leaf_names_per_element[element]:
        elements_list.write(leaf + '\n')
        if 'RET' in leaf or 'PAT' in leaf or 'BAS' in leaf:
            DB_elements_list.write(leaf + '\n')
    elements_list.write('\n')
    DB_elements_list.write('\n')
elements_list.close()
DB_elements_list.close()

#Summarize and write table

elements = all_leaf_names_per_element.keys()
all_species = []
for element in elements: #count the nodes for each species
    leaves = all_leaf_names_per_element[element]
    count_elements_per_species[element] = {}
    for leaf in leaves:
        species = leaf.split('_')[0]
        if not species in all_species:
            all_species.append(species)
        if species in count_elements_per_species[element].keys():
            count_elements_per_species[element][species] += 1
        else:
            count_elements_per_species[element][species] = 1

from cogent import LoadTable
from cogent.util.table import Table

column_headings = ['species']
for el in elements:
    column_headings.append(el)
lines = []
for species in all_species:
    line = []
    line.append(species)
    for element in elements:
        if species in count_elements_per_species[element].keys():
            line.append(count_elements_per_species[element][species])
        else:
            line.append(0)
    lines.append(line)
table = Table(header = column_headings, rows = lines)
print table
table.writeToFile('element_hits_summary.csv', sep='\t')
```

```
=========================================================================
species    PAT    crypton    Ngaro    dirs1_like    pat1_like    kangaroo
-------------------------------------------------------------------------
   Vcar     70          0        0             0           17          24
   Acas     10          0        0            11            0           0
    BAS      0          7        7           120            3           0
    Nvi      0          1        0            46            0           0
    Nve      0          1      114            59            3           0
    Lgi      0          0       53            94            8           0
   Hduj      0          0       14             3            0           0
   Ebre      0          0        2             7            0           0
   Dpul      0          0        1           182            0           0
   Rcul      0          0        0            68            2           0
   Ppac      0          0        0             1           32           0
   Acal      0          0        0            33            0           0
    RET      0          0        0             5            1           0
   Hcon      0          0        0             0           18           0
    PAT      0          0        0             0            4           0
   Cele      0          0        0             0            1           0
   Cjap      0          0        0             0           50           0
   Crem      0          0        0             0           11           0
   Cang      0          0        0             0            2           0
   briC      0          0        0             0           25           0
-------------------------------------------------------------------------
```

# Figure 3: Draw the table results on the Nematoda tree¶

In [4]:

```
from ete2 import Tree, NodeStyle, TreeStyle, TextFace, PhyloTree,PieChartFace, CircleFace, BarChartFace 
from cogent import LoadTable
from cogent.util.table import Table
import numpy

Nematoda_tree = Tree('Fitch2013_Adapted_DeLey2006.tre', format = 1)
Nematoda_tree.ladderize(1)

ns = NodeStyle()
ns['hz_line_width']=9
ns['vt_line_width']=9
ns["shape"]='square'
ns["fgcolor"] = "black"
ns["size"] = 9
    
for node in Nematoda_tree.traverse():
    node.set_style(ns)
    

hits_table = LoadTable(filename = 'element_hits_summary.csv', header = True, with_title = False, sep = '\t')
species = []
for row in hits_table:
    species.append(row['species'])
elements = hits_table._header
elements.pop(0)

nematode_species_table =LoadTable(filename = 'nematode_species.csv', header = True, with_title = False, sep = '\t')

colors = {
          'pat1_like': 'blue',
          'dirs1_like': 'darkviolet',
          'PAT': 'green',
          'kangaroo': 'red',
          'Ngaro':'black',
          'crypton': 'white',
          }

    
color_list = []
for el in elements:
    color_list.append(colors[el])

for leaf in Nematoda_tree:
    hola = TextFace(leaf.name + '   ', fsize = 50)
    leaf.add_face(hola, column=0, position = "branch-right")        
    leaf.add_features(species = {}, total_counts_per_speices = {}, per_element_counts_per_species = {},  per_element_percents_per_species = {})
    for row in nematode_species_table:
        if row['LowerRank'] == leaf.name:
            leaf.species[row['Abbreviation']] = row['Species']
    for row in hits_table:
        if row['species'] in leaf.species.keys():
            counts = []
            for i in row[1:]:
                counts.append(i)
            total_count = sum(counts)
            percents = []
            for count in counts:
                percent = float(count)/total_count*100
                percents.append(percent)
            leaf.total_counts_per_speices[row['species']] = total_count
            leaf.per_element_counts_per_species[row['species']] = counts
            leaf.per_element_percents_per_species[row['species']] = percents
    species_num = 1
    code_list = leaf.species.keys()
    if leaf.name == 'Rhabditomorpha':
        code_list = ['Cele', 'briC', 'Cjap', 'Crem', 'Cbre', 'C5sp', 'C11sp', 'Otip', 'Hbac', 'Hcon', 'Dviv'] # to order by taxonomy
    for sp in code_list:
        hola = TextFace(leaf.species[sp].split(' ')[0][0]+ '. ' + leaf.species[sp].split(' ')[1] + ' ', fsize = 55, tight_text=False, fstyle='italic')
        hola.margin_bottom=35
        hola.margin_bottom=35
        leaf.add_face(hola, column=1, position = "aligned")
        if sp in leaf.total_counts_per_speices.keys():
            size = 30
            if leaf.total_counts_per_speices[sp] > 10 and leaf.total_counts_per_speices[sp] < 190:
                size = leaf.total_counts_per_speices[sp]*3
            elif leaf.total_counts_per_speices[sp] >=  190:
                size = 571
            count_face = TextFace(str(leaf.total_counts_per_speices[sp])+' ', fsize = 50, fgcolor = 'black')
            count_face.margin_bottom=30
            count_face.margin_bottom=30
            leaf.add_face(count_face, column=2, position = "aligned")
            pie = PieChartFace(leaf.per_element_percents_per_species[sp], size, size, color_list, line_color='black')
            max_val = 5 # y axis maximum for bar chart faces
            m = max(leaf.per_element_counts_per_species[sp])
            if m > max_val and m < 100:
                max_val = 100
            elif m > max_val:
                max_val = m
            #max_val = m
            bar = BarChartFace(leaf.per_element_counts_per_species[sp], colors=color_list, min_value=0, max_value=max_val, width=260, height=130,)
            if not leaf in (Nematoda_tree&'Nematoda'): 
                pie.margin_bottom=65
                pie.margin_top=65
                bar.margin_bottom=60
                bar.margin_top=60
            leaf.add_face(pie, column=3, position = "aligned")
            #leaf.add_face(bar, column=3, position = "aligned")
        else:
            count_face = TextFace('0 ', fsize = 50, fgcolor = 'black')
            count_face.margin_bottom=35
            count_face.margin_bottom=35
            leaf.add_face(count_face, column=2, position = "aligned")
            dummy = CircleFace(0, color = 'black')
            dummy.margin_bottom=35
            dummy.margin_bottom=35
            leaf.add_face(dummy, column=3, position = "aligned")
        species_num += 1


for n in Nematoda_tree.traverse():
    if not n.is_leaf() and n.name == 'Nematoda':
        hola = TextFace(n.name, fsize = 50, fgcolor = 'black')
        if n.name == 'Nematoda':
            nst = NodeStyle()
            nst['hz_line_width']=9
            nst['vt_line_width']=9
            n.set_style(nst)
            
        n.add_face(hola, column=0, position = "branch-right") 
            
Nematoda_tree.write(outfile='./Fitch2013_Adapted_DeLey2006.nhx', format=1, features=["species", "total_counts_per_speices", "per_element_counts_per_species", "per_element_percents_per_species"])

Nematoda_tree.convert_to_ultrametric(10)

legend = [
          ['PAT and Kangaroo', 'green'],
          ['pat1_like', 'blue'],
          ['dirs1_like', 'darkviolet'],
          ['Ngaro', 'black'],
          ['Crypton', 'white'],
          ]

tsn = TreeStyle()
pos = 0
#for item in legend:
#    tsn.legend.add_face(TextFace('  ' + item[0] + ' ', fsize=50), column=pos+1)
#    tsn.legend.add_face(CircleFace(50, item[1], style='circle'), column=pos)
#    pos += 2
tsn.scale = 10
tsn.allow_face_overlap=False
tsn.show_leaf_name = False
tsn.min_leaf_separation = 90
tsn.draw_guiding_lines = False
#tsn.legend_position=3
Nematoda_tree.dist = 0
(Nematoda_tree&'Nematoda').convert_to_ultrametric(10)


stdin = (Nematoda_tree&'Nematoda').render('Figure3_inset.tif', w=3000, tree_style = tsn)
#stdin = (Nematoda_tree&'Nematoda').render('Figure3_inset_BARS.tif', w=3000, tree_style = tsn)
for n in (Nematoda_tree&'Nematoda').get_children():
    n.detach()
Nematoda_tree.convert_to_ultrametric(10)
stdin = Nematoda_tree.render('Figure3_main.tif', w=3000, tree_style = tsn)
#stdin = Nematoda_tree.render('Figure3_main_BARS.tif', w=3000, tree_style = tsn)
```

# Make Figure S2

Which shows the association between reduced and full YR tree clades

In [6]:

```
from ete2 import Tree, TreeFace, TreeStyle, CircleFace, TextFace, NodeStyle
import re

placement = {'PAT__node235': {'full': ['node78'], 'reduced': ['node235']}, 'PAT__node236': {'full': [], 'reduced': ['node236']}, 'PAT__node237': {'full': ['node210'], 'reduced': ['node237']}, 'Crypton__node165': {'full': ['RET_CryptonCn1_1358_1_YR_only_crypton', 'node148'], 'reduced': ['node165']}, 'Ngaro__node167': {'full': ['node102', 'node82', 'BAS_DIRS_53_XT_3142_1_viper_or_Ngaro', 'node263', 'node448', 'node546'], 'reduced': ['node167']}, 'Crypton__BAS_CryptonA_1_OL_300_1_YR_only_crypton': {'full': ['BAS_CryptonA_1_OL_300_1_YR_only_crypton'], 'reduced': ['BAS_CryptonA_1_OL_300_1_YR_only_crypton']}, 'Ngaro__node222': {'full': [], 'reduced': ['node222']}, 'pat1_like': {'full': ['node224', 'node219', 'node119', 'node217'], 'reduced': ['node194', 'node197', 'node196', 'node262', 'node260', 'node325']}, 'Crypton__BAS_CryptonF_6_PI_451_1_YR_only_crypton': {'full': ['node204'], 'reduced': ['BAS_CryptonF_6_PI_451_1_YR_only_crypton']}, 'Crypton__BAS_CryptonV_1_NV_2095_1_YR_only_crypton': {'full': ['BAS_CryptonV_1_NV_2095_1_YR_only_crypton'], 'reduced': ['BAS_CryptonV_1_NV_2095_1_YR_only_crypton']}, 'PAT__node266': {'full': ['node133'], 'reduced': ['node266']}, 'PAT__node229': {'full': ['node213'], 'reduced': ['node229']}, 'Crypton__node173': {'full': ['BAS_Crypton_10_CGi_839_1_YR_only_crypton', 'BAS_Crypton_9_CGi_1469_1_YR_only_crypton', 'BAS_CryptonA_3_SK_54_1_YR_only_crypton', 'node240'], 'reduced': ['node173']}, 'PAT__node160': {'full': ['node79', 'node56'], 'reduced': ['node160']}, 'kangaroo': {'full': ['node93'], 'reduced': ['Vcar_scaffold_38_112287_1_kangaroo']}, 'Ngaro__node135': {'full': ['node102', 'node155'], 'reduced': ['node135']}, 'dirs1_like': {'full': ['node104', 'node103', 'node382', 'node98', 'node403', 'node299', 'node298', 'node293', 'node78', 'node112', 'node294', 'node381', 'node287', 'node111', 'node110', 'node109', 'node95', 'node120', 'node105', 'RET_RoDirs1_3186_1_dirs1_like', 'node373', 'BAS_DIRS_4_SK_3309_1_DIRS', 'node295', 'node107', 'node291', 'node290', 'node379', 'node376', 'node377', 'node292'], 'reduced': ['node77', 'node73', 'node17', 'node32', 'node30', 'node191', 'node193', 'node8', 'node95', 'node94', 'node50', 'node131', 'node180', 'RET_TcDirs1_3616_1_dirs1_like', 'RET_RoDirs1_3186_1_dirs1_like', 'node68', 'node46', 'node51', 'node3', 'node66', 'node161', 'node121', 'node122', 'node5', 'node4', 'node7']}, 'Crypton__BAS_Crypton_1_NV_637_1_YR_only_crypton': {'full': ['node683'], 'reduced': ['BAS_Crypton_1_NV_637_1_YR_only_crypton']}, 'PAT__node192': {'full': ['node90'], 'reduced': ['node192']}, 'Crypton__BAS_CryptonA_1_LG_614_1_two_domains_crypton': {'full': ['BAS_CryptonA_1_LG_614_1_two_domains_crypton'], 'reduced': ['BAS_CryptonA_1_LG_614_1_two_domains_crypton']}, 'Crypton__node134': {'full': ['node215'], 'reduced': ['node134']}, 'Crypton__BAS_CryptonS_2_PU_272_1_YR_only_crypton': {'full': ['BAS_CryptonS_2_PU_272_1_YR_only_crypton'], 'reduced': ['BAS_CryptonS_2_PU_272_1_YR_only_crypton']}, 'Crypton__BAS_Crypton_1_NV_309_1_YR_only_crypton': {'full': [], 'reduced': ['BAS_Crypton_1_NV_309_1_YR_only_crypton']}}


# This is the unedited dictionary

full_tree = Tree('YR_tree/all_YR_corrected_reviewed_internal_nodes.tre', format=1)
full_tree_descendent_names = []
for node in full_tree.get_descendants():
    full_tree_descendent_names.append(node.name)
reduced_tree = Tree('comparable_trees/YR_outgrouped_corrected_reviewed_internal_nodes.tre', format=1)
reduced_tree_descendent_names = []
for node in reduced_tree.get_descendants():
    reduced_tree_descendent_names.append(node.name)


def add_node_support(node):
    if node.support == 1:
         node.add_face(CircleFace(radius = 4, color = 'black'),column=0, position = "float")
    elif node.support < 1 and node.support >= 0.95:
         node.add_face(CircleFace(radius = 4, color = 'gray'),column=0, position = "float")
    elif node.support < 0.95 and node.support >= 0.9:
         node.add_face(CircleFace(radius = 4, color = 'gray'),column=0, position = "float")
    elif node.support < 0.9 and node.support >= 0.8:
         node.add_face(CircleFace(radius = 4, color = 'gainsboro'),column=0, position = "float")

def add_leaf_name(leaf,color,size):
    name = leaf.name
    pattern = re.search(r'(^[^_]+)_.+_(\d+)_1_(.+)', name)
    new_name = pattern.group(1) + ' ' + pattern.group(2) + ' ' + pattern.group(3)
    leaf_name = TextFace(new_name,fsize=size, fgcolor=color)
    leaf.add_face(leaf_name,0,position='branch-right')
    
def find_identical_leaves_between_clades_of_different_trees(node_a,node_b):
    matching_leaf_names = []
    leaves_a = node_a.get_leaf_names()
    leaves_b = node_b.get_leaf_names()
    matching_leaf_names = list(set(leaves_a).intersection(leaves_b))
    return matching_leaf_names


reduced_tree_leaves_that_were_found_in_the_full_tree = []

ts = TreeStyle()
ts.draw_guiding_lines = False
ts.show_leaf_name = False
ts.scale = 50

ns = NodeStyle(hz_line_color = 'black', vt_line_color = 'black', hz_line_width = 3, vt_line_width = 3, fgcolor = 'black', size = 0)    

ts2 = TreeStyle()
ts2.draw_guiding_lines = False
ts2.show_leaf_name = False
ts2.scale = 5


for group_name in placement.keys():
    reduced_tree_clades = []
    full_tree_clades = []
    if 'reduced' in placement[group_name].keys() and 'full' in placement[group_name].keys():
        reduced_tree_clades = placement[group_name]['reduced']
        full_tree_clades = placement[group_name]['full']
    full_tree = Tree('YR_tree/all_YR_corrected_reviewed_internal_nodes.tre', format=1)
    if len(full_tree_clades) > 0 and len(reduced_tree_clades) > 0:
        for full_tree_clade in full_tree_clades:
            if full_tree_clade in full_tree_descendent_names:
                found = 0
                for reduced_tree_clade in reduced_tree_clades:
                    if found == 0 and reduced_tree_clade in reduced_tree_descendent_names:
                        matches = find_identical_leaves_between_clades_of_different_trees(full_tree&full_tree_clade,reduced_tree&reduced_tree_clade)
                        if len(matches) > 0:
                            found = 1
                            reduced_tree_leaves_that_were_found_in_the_full_tree = reduced_tree_leaves_that_were_found_in_the_full_tree + matches
                            full_tree = Tree('YR_tree/all_YR_corrected_reviewed_internal_nodes.tre', format=1)
                            (full_tree&full_tree_clade).dist = 0
                            Nstyle = NodeStyle(hz_line_color = 'black', vt_line_color = 'black',
                                               hz_line_width = 3, vt_line_width = 3, fgcolor = 'black', size = 0)
                            Nstyle["bgcolor"] = 'gainsboro'
                            (full_tree&full_tree_clade).set_style(Nstyle)
                            for n in (full_tree&full_tree_clade).traverse():
                                n.set_style(Nstyle)
                            for leaf in full_tree&full_tree_clade:
                                if leaf.name in matches:
                                    add_leaf_name(leaf,'black',15)
                                else:
                                    add_leaf_name(leaf,'black',9)
                            add_node_support(full_tree&full_tree_clade)
                            clade_face = TreeFace(full_tree&full_tree_clade, ts2)
                            clade_face_newick = (full_tree&full_tree_clade).write()
                            (reduced_tree&matches[0]).add_face(clade_face, 4, position = 'aligned')
                            (reduced_tree&matches[0]).add_feature('full_tree_node',clade_face_newick)
                            

for leaf in reduced_tree:
    if leaf.name in reduced_tree_leaves_that_were_found_in_the_full_tree and 'RET' in leaf.name or 'BAS' in leaf.name or 'PAT ' in leaf.name:
        add_leaf_name(leaf,'green',15)
    elif leaf.name in reduced_tree_leaves_that_were_found_in_the_full_tree:
        add_leaf_name(leaf,'black',15)
    elif 'RET' in leaf.name or 'BAS' in leaf.name or 'PAT ' in leaf.name:
        add_leaf_name(leaf,'black',15)
    else:
        add_leaf_name(leaf,'black',9)
                

reduced_tree.ladderize()
R = reduced_tree.get_midpoint_outgroup()
reduced_tree.set_outgroup(R)
for node in reduced_tree.traverse():
    if not node.is_leaf():
        add_node_support(node)
for n in reduced_tree.traverse():
    n.set_style(ns)
reduced_tree.set_style(ns)
#reduced_tree.show(tree_style = ts)
print 'rendering figure'

ts.legend_position=1
ts.legend.add_face(TextFace('sh-like support: ', fsize=20), column=0)
ts.legend.add_face(CircleFace(radius = 8, color = 'black'), column=1)
ts.legend.add_face(TextFace(' 1 ', fsize=20), column=2)
ts.legend.add_face(CircleFace(radius = 8, color = 'gray'), column=3)
ts.legend.add_face(TextFace(' 0.9-1 ', fsize=20), column=4)
ts.legend.add_face(CircleFace(radius = 8, color = 'gainsboro'), column=5)
ts.legend.add_face(TextFace(' 0.8-0.9 ', fsize=20), column=6)
stdout = reduced_tree.render('./FigureS2.pdf', w=1000, tree_style = ts)
reduced_tree.write(features=[], outfile='phylogenetic_classification.nhx')
```

```
rendering figure
```

# Make Figure 2 a, the collapsed RT tree

In [9]:

```
from ete2 import Tree, NodeStyle, TreeStyle, TextFace, PhyloTree, CircleFace, PieChartFace
import sys, numpy
path = './'

def blen_cutoff(node,brlns_cutoff_factor):
    blens = []
    for br in node.traverse():
        if not br.name == node.name and not br.dist == 0:
            blens.append(float(br.dist))
    return numpy.median(blens)*brlns_cutoff_factor


tree_string_files = [
                     #'YR_tree/all_YR_corrected_reviewed.tre',
                     #'comparable_trees/RT_corrected_reviewed.tre',
                     'comparable_trees/RT_outgrouped_corrected_reviewed.tre',
                     #'comparable_trees/RTYR_corrected_reviewed.tre',
                     #'comparable_trees/YR_corrected_reviewed.tre',
                     #'comparable_trees/YR_outgrouped_corrected_reviewed.tre'
                    ]
   


for tree_string_file in tree_string_files:
    support_cutoff = 0.01
    #if tree_string_file ==  'YR_tree/all_YR_corrected_reviewed.tre':
    #    support_cutoff = 0.9 #higher cutoff for phylo-placing than for just phylo presenting
    #elif tree_string_file ==  'comparable_trees/YR_outgrouped_corrected_reviewed.tre':
    #    support_cutoff = 0.7 #higher cutoff for phylo-placing than for just phylo presenting
    
        
    tree_string = open(path + tree_string_file, 'r').read()
    t = Tree(tree_string, format=1)
    print len(t)
    node_number = 1 #We are going to give running numbers to all the nodes for easier access later on
    
    for n in t.traverse():
        if n.is_leaf and 'rypton' in n.name:
            n.name = n.name + '_crypton' # This will make the annotation easier
        if n.support < support_cutoff:
            n.delete()                   # multifrucate unsupported bifrucarions
            
    print_collapsed_nodes = 0            # will make a table of the collaped nodes, named by running numbers, and their 
                                         # leaf content
    
    for n in t.traverse():
        if not n.is_leaf():
            organisms_in_node = {       # will be used to count taxa in collapsed nodes     
                    'PAT': 0,
                    'BAS': 0,
                    'RET': 0,
                    'Mflo': 0,
                    'Dviv': 0,
                    'Ebre': 0,
                    'Rcul': 0,
                    'Avit': 0,
                    'Asuu': 0,
                    'Bxyl': 0,
                    'Hbac': 0,
                    'Haor': 0,
                    'Hcon': 0,
                    'Srat': 0,
                    'briC': 0,
                    'Cele': 0,
                    'Cjap': 0,
                    'Bmal': 0,
                    'Cbre': 0,
                    'Crem': 0,
                    'Gpal': 0,
                    'Lsig': 0,
                    'Ppac': 0,
                    'Lloa': 0,
                    'Mhap': 0,
                    'Minc': 0,
                    'C5sp': 0,
                    'Dimm': 0,
                    'Ooch': 0,
                    'Ovol': 0,
                    'Otip': 0,
                    'Pred': 0,
                    'C11sp': 0,
                    'Cjap': 0,
                    'Cang': 0,
                    'Tmur': 0,
                    'Tspi': 0,
                    'Wban': 0,
                    'Acas': 0,
                    'Agam': 0,
                    'Acal': 0,
                    'Dpul': 0,
                    'Dsim': 0,
                    'Lgi': 0,
                    'Nvi': 0,
                    'Nve': 0,
                    'Ath': 0,
                    'Vcar': 0,
                    'Alyr': 0,
                    'Hduj': 0
                    }
            elements_in_node = {     #will be used to count element types in collapsed nodes
                    'dirs1_like': 0,
                    'pat1_like': 0,
                    'PAT': 0,
                    'viper_or_Ngaro': 0,
                    'kangaroo': 0,
                    'DIRS': 0,
                    'domains_inverted': 0,
                    'two_domains': 0,
                    'YR_only': 0,
                    'crypton': 0,
                    'DERIVED': 0,    
                    'GYPSY': 0,
                    'COPIA': 0,
                    'BEL': 0
                    }
            # node will be collapsed only if all the leafs can be placed in only one of these arrays
            dirs1_hirarchy = ('dirs1_like', 'DIRS', 'two_domains', 'YR_only','DERIVED')
            pat_like_hirarchy = ('pat1_like', 'DIRS', 'two_domains', 'YR_only','DERIVED')
            toc_hirarchy = ('PAT', 'DIRS', 'two_domains', 'YR_only','DERIVED')
            viper_or_Ngaro_hirarchy = ('viper_or_Ngaro', 'two_domains', 'YR_only','DERIVED')
            kangaroo_hirarchy = ('kangaroo', 'domains_inverted','two_domains', 'YR_only','DERIVED', 'DIRS')
            DIRS_hirarchy = ('DIRS', 'two_domains', 'YR_only','DERIVED')
            domains_inverted_hirarchy = ('domains_inverted','two_domains', 'YR_only','DERIVED')
            two_domains_hirarchy = ('two_domains','domains_inverted', 'YR_only','DERIVED')
            crypton_hirarchy = ('crypton', 'YR_only')
            YR_hirarchy = crypton_hirarchy 
            GYPSY_hirarchy = ('GYPSY')
            COPIA_hirarchy = ('COPIA')
            BEL_hirarchy = ('BEL')
        
        
            n.name = 'node' + str(node_number) #naming the node
            node_number += 1
        
            # prepare species counts and element counts to be added to all nodes
            for leaf in n:
                    for orgn in organisms_in_node.keys(): # orgn is the species key
                        search_length = len(orgn)
                        if leaf.name[:search_length] == orgn: # if begining of the leaf name same as orgn
                            organisms_in_node[orgn] += 1      # count it
                    for element in elements_in_node.keys():   
                        search_length = len(element)                    
                        if leaf.name[-1*search_length:] == element:  # if end of the leaf name same as element
                            elements_in_node[element] += 1           # count it 
            for orgn in organisms_in_node.keys(): # get read of species codes with zero count 
                if organisms_in_node[orgn] == 0:
                    organisms_in_node.pop(orgn, None) 
            for element in elements_in_node.keys(): # get read of elements with zero count
                if elements_in_node[element] == 0:
                    elements_in_node.pop(element, None)
            n.add_features(species=organisms_in_node, elements=elements_in_node) # add speceis count and element counts
                                                                                 # as node features
    # write tree with node names for future reference        
    #.write(outfile=path + tree_string_file.split('.')[0] + '_internal_nodes.tre', format=1, features=["support"])
    #collapsed_nodes_file = open(path + tree_string_file.split('.')[0] + '_collapsed_nodes.txt','wt')
    collapsed_nodes = []
    

    # decside which node to collapse based on the presence absence of conflicting element forms
    for n in t.traverse():
        if not n.is_leaf():
            collapse = 1

            if 'dirs1_like' in n.elements.keys():
                for name in n.elements.keys():
                    if not name in dirs1_hirarchy:
                        collapse = 0
            elif 'pat1_like' in n.elements.keys():
                for name in n.elements.keys():
                    if not name in pat_like_hirarchy:
                        collapse = 0 
            elif 'PAT' in n.elements.keys():
                for name in n.elements.keys():
                    if not name in toc_hirarchy:
                        collapse = 0
            elif 'kangaroo' in n.elements.keys():
                for name in n.elements.keys():
                    if not name in kangaroo_hirarchy:
                        collapse = 0
            elif 'viper_or_Ngaro' in n.elements.keys():
                for name in n.elements.keys():
                    if not name in viper_or_Ngaro_hirarchy:
                        collapse = 0
            elif 'DIRS' in n.elements.keys():
                for name in n.elements.keys():
                    if not name in DIRS_hirarchy:
                        collapse = 0
            elif 'domains_inverted' in n.elements.keys():
                for name in n.elements.keys():
                    if not name in domains_inverted_hirarchy:
                        collapse = 0
            elif 'two_domains' in n.elements.keys():
                for name in n.elements.keys():
                    if not name in two_domains_hirarchy:
                        collapse = 0
            elif 'COPIA' in n.elements.keys():
                for name in n.elements.keys():
                    if not name in COPIA_hirarchy:
                        collapse = 0
            elif 'BEL' in n.elements.keys():
                for name in n.elements.keys():
                    if not name in BEL_hirarchy:
                        collapse = 0
            elif 'GYPSY' in n.elements.keys():
                for name in n.elements.keys():
                    if not name in GYPSY_hirarchy:
                        collapse = 0
            elif 'crypton' in n.elements.keys():
                for name in n.elements.keys():
                    if not name in crypton_hirarchy:
                        collapse = 0
            elif 'YR_only' in n.elements.keys():
                for name in n.elements.keys():
                    if not name in YR_hirarchy:
                        collapse = 0
            if collapse == 1:
                if tree_string_file == 'YR_tree/all_YR_corrected_reviewed.tre' and n.support < 0.95:
                    n_descendants = n.get_descendants()
                    cutoff = blen_cutoff(n,4)
                    for d in n_descendants:
                         if d.dist > cutoff:
                             d.detach()
                #if print_collapsed_nodes == 1: #print all leaves in a file. It will be used later 
                    #for leaf in n:
                        #if not 'node' in leaf.name:
                            #collapsed_nodes.append(leaf.name + '\t' + n.name + '\t')
                for C in n.get_descendants(): # and remove their leaves
                        C.detach() 
        
    
    
    
    ts = TreeStyle()
    ts.show_leaf_name = False
    ts.scale = 100
    ts.show_branch_support = False
    t.ladderize(1)
    R = t.get_midpoint_outgroup()
    ns = NodeStyle()
    ns['hz_line_width'] = 5
    ns['vt_line_width'] = 5
    ns['fgcolor'] = 'black'
    ns['size'] = 0
    
    
    t.set_outgroup(R)

    for node in t.traverse(): 
        node.set_style(ns)
        if node.is_leaf():           
            if not 'node' in node.name:    #add faces to leaves
                color = 'gray'
                if 'rypton' in node.name:
                    color = 'sandybrown'
                    #collapsed_nodes_file.write(node.name + '\t' + node.name + '\t' + 'Crypton' + '\n')
                elif 'kangaroo' in node.name:
                    color = 'red'
                    #collapsed_nodes_file.write(node.name + '\t' + node.name + '\t' + 'kangaroo' + '\n')
                elif 'dirs1' in node.name:
                    color = 'darkviolet'
                elif 'PAT' in node.name:
                    color = 'green'
                elif 'Ngaro' in node.name:
                    color = 'black'
                    #if ('BAS' in node.name or 'RET' in node.name) and not 'DIRS' in node.name:
                        #collapsed_nodes_file.write(node.name + '\t' + node.name + '\t' + 'Ngaro' + '\n')
                elif 'ypsy' in node.name:
                    node.name = 'GYPSY'
                    color = 'black'
                name_parts = node.name.split('_')
                name = name_parts[0]
                get_name_part = 0
                for part in name_parts:
                    if get_name_part == 1:
                        name = name + '_' + part
                    elif part == '1':
                        get_name_part = 1
                hola = TextFace(name, fsize=15, fgcolor=color)    
                #node.add_face(hola, column=0, position = "branch-right")
                if node.name == 'GYPSY':
                    node.add_face(hola, column=0, position = "branch-right")
                elif color == 'gray':
                    node.add_face(CircleFace(radius = 10, color = 'gray', style = 'sphere'), column=0, position = "branch-right")
                else:
                    node.add_face(CircleFace(radius = 10, color = color), column=0, position = "branch-right")
                
                
                #if 'RET_' in node.name or 'PAT_' in node.name or 'BAS_' in node.name:
                    #if 'dirs1_like' in node.name:
                        #collapsed_nodes_file.write(node.name + '\t' + node.name + '\t' + 'dirs1_like' + '\n')
                    #elif 'pat1_like' in node.name:
                        #collapsed_nodes_file.write(node.name + '\t' + node.name + '\t' + 'pat1_like' + '\n')
                    #elif '_PAT' in node.name:
                        #collapsed_nodes_file.write(node.name + '\t' + node.name + '\t' + 'PAT' + '\n')
                    #else:
                        #collapsed_nodes_file.write(node.name + '\t' + node.name + '\t' + 'None' + '\n')
            elif 'node' in node.name:  # add faces to collapsed nodes
                bcolor = 'white'
                species = ''
                count = 0
                for key in node.species.keys():
                    species =species + key + '_' + str(node.species[key]) + '_'
                    count += node.species[key]
                elements = ''
                for key in node.elements.keys():
                    elements = elements + key + '_' + str(node.elements[key]) + ' '
                verdict = 'None'
                verdict_color = 'black'
                if 'dirs1_like' in elements:
                    verdict = 'dirs1_like'
                    verdict_color = 'darkviolet'
                elif 'pat1_like' in elements:
                    verdict = 'pat1_like'
                    verdict_color = 'blue'
                elif 'PAT' in elements:
                    verdict = 'PAT'
                    verdict_color = 'green'
                elif 'kangaroo' in elements:
                    verdict = 'kangaroo'
                    verdict_color = 'red'
                elif 'Ngaro' in elements:
                    verdict = 'Ngaro'
                    verdict_color = 'black'
                elif 'crypton' in elements:
                    verdict = 'Crypton'
                    verdict_color = 'sandybrown'
                elif 'BEL' in elements:
                    verdict = 'BEL'
                    verdict_color = 'black'
                elif 'COPIA' in elements:
                    verdict = 'COPIA'
                    verdict_color = 'black'
                elif 'GYPSY' in elements:
                    verdict = 'GYPSY'
                    verdict_color = 'black'
                #for line in collapsed_nodes:
                    #if line.split('\t')[1] == node.name:
                        #collapsed_nodes_file.write(line + verdict + '\n')
                el = TextFace(elements, fsize=10, fgcolor = 'dimgray')
                if bcolor == 'black':
                    el.fgcolor = 'white'
                sp = TextFace(species, fsize=10, fgcolor = 'dimgray')
                if count < 15:
                    count = 15
                #if len(species) > 0:
                    #species_line_to_pie_chart_face(node, species,species_groupings,float(count)*1+5)
                #sp.border.width = 1
                
                vr = TextFace(verdict, fsize=count, fgcolor=verdict_color)
                #if verdict == 'pat1_like':
                    #vr.background.color = 'lightgray'
                #node.add_face(sp, column=2, position = "branch-right")
                
                #node.add_face(el, column=2, position = "branch-right")
                if not verdict == 'None' and not verdict == 'COPIA' and not verdict == 'BEL' and not verdict == 'GYPSY':
                    node.add_face(CircleFace(radius = 10, color = verdict_color), column=0, position = "branch-right")
                    #node.add_face(vr, column=1, position = "branch-right")
                   
                elif not verdict == 'COPIA' and not verdict == 'BEL' and not verdict == 'GYPSY':
                    node.add_face(CircleFace(radius = 10, color = 'gray', style = 'sphere'), column=0, position = "branch-right")
                else:
                    node.add_face(vr, column=1, position = "branch-right")
                hola = TextFace(node.name, fsize=10, fgcolor='gray')    
                #node.add_face(hola, column=0, position = "branch-right")
                score = TextFace(str(node.support),fsize=6, fgcolor='black')
                #node.add_face(score,column=0, position = "branch-right")
                if node.support == 1:
                    node.add_face(CircleFace(radius = 5, color = 'black'),column=0, position = "float")
                elif node.support < 1 and node.support >= 0.95:
                    node.add_face(CircleFace(radius = 5, color = 'dimgray'),column=0, position = "float")
                elif node.support < 0.95 and node.support >= 0.9:
                    node.add_face(CircleFace(radius = 5, color = 'gray'),column=0, position = "float")
                elif node.support < 0.9 and node.support >= 0.8:
                    node.add_face(CircleFace(radius = 5, color = 'gainsboro'),column=0, position = "float")
        else: #add faces to internal nodes
            hola = TextFace(node.name, fsize=10, fgcolor='gray')    
            #node.add_face(hola, column=0, position = "branch-right")
            if node.support == 1:
                    node.add_face(CircleFace(radius = 5, color = 'black'),column=0, position = "float")
            elif node.support < 1 and node.support >= 0.95:
                    node.add_face(CircleFace(radius = 5, color = 'dimgray'),column=0, position = "float")
            elif node.support < 0.95 and node.support >= 0.9:
                    node.add_face(CircleFace(radius = 5, color = 'gray'),column=0, position = "float")
            elif node.support < 0.9 and node.support >= 0.8:
                    node.add_face(CircleFace(radius = 5, color = 'gainsboro'),column=0, position = "float")
            
    
    t.dist = 0
    t.support = 0
    #t.show(tree_style = ts)
    print 'rendering ' + tree_string_file
    t.render(path + tree_string_file.split('.')[0] + '_Figure.tif', w = 1000, tree_style = ts)
    #collapsed_nodes_file.close()
```

```
520
rendering comparable_trees/RT_outgrouped_corrected_reviewed.tre
```

# Make Figure 2b, the collapsed reduced YR tree

In [19]:

```
from ete2 import Tree, NodeStyle, TreeStyle, TextFace, PhyloTree, CircleFace, PieChartFace
import sys, numpy
path = './'


def blen_cutoff(node,brlns_cutoff_factor):
    blens = []
    for br in node.traverse():
        if not br.name == node.name and not br.dist == 0:
            blens.append(float(br.dist))
    return numpy.median(blens)*brlns_cutoff_factor


tree_string_files = [
                    #'YR_tree/all_YR_corrected_reviewed.tre',
                    #'comparable_trees/RT_corrected_reviewed.tre',
                    #'comparable_trees/RT_outgrouped_corrected_reviewed.tre',
                    #'comparable_trees/RTYR_corrected_reviewed.tre',
                    #'comparable_trees/YR_corrected_reviewed.tre',
                    'comparable_trees/YR_outgrouped_corrected_reviewed.tre'
                    ]


def median_blens(node):
    blens = []
    for br in node.traverse():
        blens.append(br.dist)
    return 0, numpy.median(blens), numpy.median(blens)*2 # type median_blens(node)[2] to get median brln times 2
    


for tree_string_file in tree_string_files:
    support_cutoff = 0.01
#    if tree_string_file ==  'YR_tree/all_YR_corrected_reviewed.tre':
#        support_cutoff = 0.8 #higher cutoff for phylo-placing than for just phylo presenting
#    elif tree_string_file ==  'comparable_trees/YR_outgrouped_corrected_reviewed.tre':
#        support_cutoff = 0.7 #higher cutoff for phylo-placing than for just phylo presenting
    
        
    tree_string = open(path + tree_string_file, 'r').read()
    t = Tree(tree_string, format=1)
    print len(t)
    node_number = 1 #We are going to give running numbers to all the nodes for easier access later on
    
    for n in t.traverse():
        if n.is_leaf and 'rypton' in n.name:
            n.name = n.name + '_crypton' # This will make the annotation easier
        if n.support < support_cutoff:
            n.delete()                   # multifrucate unsupported bifrucarions
            
    print_collapsed_nodes = 0            # will make a table of the collaped nodes, named by running numbers, and their 
                                         # leaf content
    
    for n in t.traverse():
        if not n.is_leaf():
            organisms_in_node = {       # will be used to count taxa in collapsed nodes     
                    'PAT': 0,
                    'BAS': 0,
                    'RET': 0,
                    'Mflo': 0,
                    'Dviv': 0,
                    'Ebre': 0,
                    'Rcul': 0,
                    'Avit': 0,
                    'Asuu': 0,
                    'Bxyl': 0,
                    'Hbac': 0,
                    'Haor': 0,
                    'Hcon': 0,
                    'Srat': 0,
                    'briC': 0,
                    'Cele': 0,
                    'Cjap': 0,
                    'Bmal': 0,
                    'Cbre': 0,
                    'Crem': 0,
                    'Gpal': 0,
                    'Lsig': 0,
                    'Ppac': 0,
                    'Lloa': 0,
                    'Mhap': 0,
                    'Minc': 0,
                    'C5sp': 0,
                    'Dimm': 0,
                    'Ooch': 0,
                    'Ovol': 0,
                    'Otip': 0,
                    'Pred': 0,
                    'C11sp': 0,
                    'Cjap': 0,
                    'Cang': 0,
                    'Tmur': 0,
                    'Tspi': 0,
                    'Wban': 0,
                    'Acas': 0,
                    'Agam': 0,
                    'Acal': 0,
                    'Dpul': 0,
                    'Dsim': 0,
                    'Lgi': 0,
                    'Nvi': 0,
                    'Nve': 0,
                    'Ath': 0,
                    'Vcar': 0,
                    'Alyr': 0,
                    'Hduj': 0
                    }
            elements_in_node = {     #will be used to count element types in collapsed nodes
                    'dirs1_like': 0,
                    'pat1_like': 0,
                    'PAT': 0,
                    'viper_or_Ngaro': 0,
                    'kangaroo': 0,
                    'DIRS': 0,
                    'domains_inverted': 0,
                    'two_domains': 0,
                    'YR_only': 0,
                    'crypton': 0,
                    'DERIVED': 0,    
                    'GYPSY': 0,
                    'COPIA': 0,
                    'BEL': 0
                    }
            # node will be collapsed only if all the leafs can be placed in only one of these arrays
            dirs1_hirarchy = ('dirs1_like', 'DIRS', 'two_domains', 'YR_only','DERIVED')
            pat_like_hirarchy = ('pat1_like', 'DIRS', 'two_domains', 'YR_only','DERIVED')
            toc_hirarchy = ('PAT', 'DIRS', 'two_domains', 'YR_only','DERIVED')
            viper_or_Ngaro_hirarchy = ('viper_or_Ngaro', 'two_domains', 'YR_only','DERIVED')
            kangaroo_hirarchy = ('kangaroo', 'domains_inverted','two_domains', 'YR_only','DERIVED', 'DIRS')
            DIRS_hirarchy = ('DIRS', 'two_domains', 'YR_only','DERIVED')
            domains_inverted_hirarchy = ('domains_inverted','two_domains', 'YR_only','DERIVED')
            two_domains_hirarchy = ('two_domains','domains_inverted', 'YR_only','DERIVED')
            crypton_hirarchy = ('crypton', 'YR_only')
            YR_hirarchy = crypton_hirarchy 
            GYPSY_hirarchy = ('GYPSY')
            COPIA_hirarchy = ('COPIA')
            BEL_hirarchy = ('BEL')
        
        
            n.name = 'node' + str(node_number) #naming the node
            node_number += 1
        
            # prepare species counts and element counts to be added to all nodes
            for leaf in n:
                    for orgn in organisms_in_node.keys(): # orgn is the species key
                        search_length = len(orgn)
                        if leaf.name[:search_length] == orgn: # if begining of the leaf name same as orgn
                            organisms_in_node[orgn] += 1      # count it
                    for element in elements_in_node.keys():   
                        search_length = len(element)                    
                        if leaf.name[-1*search_length:] == element:  # if begining of the leaf name same as orgn
                            elements_in_node[element] += 1           # count it 
            for orgn in organisms_in_node.keys(): # get read of species codes with zero count 
                if organisms_in_node[orgn] == 0:
                    organisms_in_node.pop(orgn, None) 
            for element in elements_in_node.keys(): # get read of elements with zero count
                if elements_in_node[element] == 0:
                    elements_in_node.pop(element, None)
            n.add_features(species=organisms_in_node, elements=elements_in_node) # add speceis count and element counts
                                                                                 # as node features
    # write tree with node names for future reference        
    #t.write(outfile=path + tree_string_file.split('.')[0] + '_internal_nodes.tre', format=1, features=["support"])
    #collapsed_nodes_file = open(path + tree_string_file.split('.')[0] + '_delete_this.txt','wt')
    collapsed_nodes = []
    

    # decside which node to collapse based on the presence absence of conflicting element forms
    for n in t.traverse():
        if not n.is_leaf():
            collapse = 1

            if 'dirs1_like' in n.elements.keys():
                for name in n.elements.keys():
                    if not name in dirs1_hirarchy:
                        collapse = 0
            elif 'pat1_like' in n.elements.keys():
                for name in n.elements.keys():
                    if not name in pat_like_hirarchy:
                        collapse = 0 
            elif 'PAT' in n.elements.keys():
                for name in n.elements.keys():
                    if not name in toc_hirarchy:
                        collapse = 0
            elif 'kangaroo' in n.elements.keys():
                for name in n.elements.keys():
                    if not name in kangaroo_hirarchy:
                        collapse = 0
            elif 'viper_or_Ngaro' in n.elements.keys():
                for name in n.elements.keys():
                    if not name in viper_or_Ngaro_hirarchy:
                        collapse = 0
            elif 'DIRS' in n.elements.keys():
                for name in n.elements.keys():
                    if not name in DIRS_hirarchy:
                        collapse = 0
            elif 'domains_inverted' in n.elements.keys():
                for name in n.elements.keys():
                    if not name in domains_inverted_hirarchy:
                        collapse = 0
            elif 'two_domains' in n.elements.keys():
                for name in n.elements.keys():
                    if not name in two_domains_hirarchy:
                        collapse = 0
            elif 'COPIA' in n.elements.keys():
                for name in n.elements.keys():
                    if not name in COPIA_hirarchy:
                        collapse = 0
            elif 'BEL' in n.elements.keys():
                for name in n.elements.keys():
                    if not name in BEL_hirarchy:
                        collapse = 0
            elif 'GYPSY' in n.elements.keys():
                for name in n.elements.keys():
                    if not name in GYPSY_hirarchy:
                        collapse = 0
            elif 'crypton' in n.elements.keys():
                for name in n.elements.keys():
                    if not name in crypton_hirarchy:
                        collapse = 0
            elif 'YR_only' in n.elements.keys():
                for name in n.elements.keys():
                    if not name in YR_hirarchy:
                        collapse = 0
            if collapse == 1:
                if tree_string_file == 'YR_tree/all_YR_corrected_reviewed.tre' and n.support < 0.95:
                    n_descendants = n.get_descendants()
                    cutoff = blen_cutoff(n,4)
                    for d in n_descendants:
                         if d.dist > cutoff:
                             d.detach()
                #if print_collapsed_nodes == 1: #print all leaves in a file. It will be used later 
                    #for leaf in n:
                        #if not 'node' in leaf.name:
                            #collapsed_nodes.append(leaf.name + '\t' + n.name + '\t')
                for C in n.get_descendants(): # and remove ther leaves
                        C.detach() 
        
    
    
    
    ts = TreeStyle()
    ts.show_leaf_name = False
    ts.scale = 10
    ts.min_leaf_separation = 8
    ts.show_branch_support = False
    t.ladderize(1)
    #R = t.get_midpoint_outgroup()
    R = t&('node254')
    ns = NodeStyle()
    ns['hz_line_width'] = 5
    ns['vt_line_width'] = 5
    ns['fgcolor'] = 'black'
    ns['size'] = 0
    
    
    t.set_outgroup(R)

    for node in t.traverse(): 
        node.set_style(ns)
        if node.is_leaf():           
            if not 'node' in node.name:
                color = 'gray'
                if 'rypton' in node.name:
                    color = 'sandybrown'
                    #collapsed_nodes_file.write(node.name + '\t' + node.name + '\t' + 'Crypton' + '\n')
                elif 'dirs1' in node.name:
                    color = 'darkviolet'
                elif 'PAT' in node.name:
                    color = 'green'
                elif 'kangaroo' in node.name:
                    color = 'red'
                elif 'Ngaro' in node.name:
                    color = 'black'
                    #if ('BAS' in node.name or 'RET' in node.name) and not 'DIRS' in node.name:
                        #collapsed_nodes_file.write(node.name + '\t' + node.name + '\t' + 'Ngaro' + '\n')
                elif 'ypsy' in node.name:
                    node.name = 'GYPSY'
                    color = 'black'
                name_parts = node.name.split('_')
                name = name_parts[0]
                get_name_part = 0
                for part in name_parts:
                    if get_name_part == 1:
                        name = name + '_' + part
                    elif part == '1':
                        get_name_part = 1
                hola = TextFace(name, fsize=15, fgcolor=color)    
                #node.add_face(hola, column=0, position = "branch-right")
                if node.name == 'GYPSY':
                    node.add_face(hola, column=0, position = "branch-right")
                elif color == 'gray':
                    node.add_face(CircleFace(radius = 10, color = 'gray', style = 'sphere'), column=0, position = "branch-right")
                else:
                    node.add_face(CircleFace(radius = 10, color = color), column=0, position = "branch-right")
                
                
                #if 'RET_' in node.name or 'PAT_' in node.name or 'BAS_' in node.name:
                    #if 'dirs1_like' in node.name:
                        #collapsed_nodes_file.write(node.name + '\t' + node.name + '\t' + 'dirs1_like' + '\n')
                    #elif 'pat1_like' in node.name:
                        #collapsed_nodes_file.write(node.name + '\t' + node.name + '\t' + 'pat1_like' + '\n')
                    #elif '_PAT' in node.name:
                        #collapsed_nodes_file.write(node.name + '\t' + node.name + '\t' + 'PAT' + '\n')
                    #else:
                        #collapsed_nodes_file.write(node.name + '\t' + node.name + '\t' + 'None' + '\n')
            elif 'node' in node.name:
                bcolor = 'white'
                species = ''
                count = 0
                for key in node.species.keys():
                    species =species + key + '_' + str(node.species[key]) + '_'
                    count += node.species[key]
                elements = ''
                for key in node.elements.keys():
                    elements = elements + key + '_' + str(node.elements[key]) + ' '
                verdict = 'None'
                verdict_color = 'black'
                if 'dirs1_like' in elements:
                    verdict = 'dirs1_like'
                    verdict_color = 'darkviolet'
                elif 'pat1_like' in elements:
                    verdict = 'pat1_like'
                    verdict_color = 'blue'
                elif 'PAT' in elements:
                    verdict = 'PAT'
                    verdict_color = 'green'
                elif 'kangaroo' in elements:
                    verdict = 'kangaroo'
                    verdict_color = 'red'
                elif 'Ngaro' in elements:
                    verdict = 'Ngaro'
                    verdict_color = 'black'
                elif 'crypton' in elements:
                    verdict = 'Crypton'
                    verdict_color = 'sandybrown'
                elif 'BEL' in elements:
                    verdict = 'BEL'
                    verdict_color = 'black'
                elif 'COPIA' in elements:
                    verdict = 'COPIA'
                    verdict_color = 'black'
                elif 'GYPSY' in elements:
                    verdict = 'GYPSY'
                    verdict_color = 'black'
                #for line in collapsed_nodes:
                    #if line.split('\t')[1] == node.name:
                        #collapsed_nodes_file.write(line + verdict + '\n')
                el = TextFace(elements, fsize=10, fgcolor = 'dimgray')
                if bcolor == 'black':
                    el.fgcolor = 'white'
                sp = TextFace(species, fsize=10, fgcolor = 'dimgray')
                if count < 15:
                    count = 15
                #if len(species) > 0:
                    #species_line_to_pie_chart_face(node, species,species_groupings,float(count)*1+5)
                #sp.border.width = 1
                
                vr = TextFace(verdict, fsize=count, fgcolor=verdict_color)
                if verdict == 'pat1_like':
                    vr.background.color = 'lightgray'
                #node.add_face(sp, column=2, position = "branch-right")
                
                #node.add_face(el, column=2, position = "branch-right")
                if not verdict == 'None' and not verdict == 'COPIA' and not verdict == 'BEL' and not verdict == 'GYPSY':
                    node.add_face(CircleFace(radius = 10, color = verdict_color), column=0, position = "branch-right")
                    #node.add_face(vr, column=1, position = "branch-right")
                   
                elif not verdict == 'COPIA' and not verdict == 'BEL' and not verdict == 'GYPSY':
                    node.add_face(CircleFace(radius = 10, color = 'gray', style = 'sphere'), column=0, position = "branch-right")
                #
                #else:
                    #node.add_face(vr, column=1, position = "branch-right")
                #hola = TextFace(node.name, fsize=10, fgcolor='gray')    
                #node.add_face(hola, column=0, position = "branch-right")
                score = TextFace(str(node.support),fsize=6, fgcolor='black')
                
                #node.add_face(score,column=0, position = "branch-right")
                if node.support == 1:
                    node.add_face(CircleFace(radius = 5, color = 'black'),column=0, position = "float")
                elif node.support < 1 and node.support >= 0.95:
                    node.add_face(CircleFace(radius = 5, color = 'dimgray'),column=0, position = "float")
                elif node.support < 0.95 and node.support >= 0.9:
                    node.add_face(CircleFace(radius = 5, color = 'gray'),column=0, position = "float")
                elif node.support < 0.9 and node.support >= 0.8:
                    node.add_face(CircleFace(radius = 5, color = 'gainsboro'),column=0, position = "float")
        else:
            #hola = TextFace(node.name, fsize=10, fgcolor='gray')    
            #node.add_face(hola, column=0, position = "branch-right")
            if node.support == 1:
                    node.add_face(CircleFace(radius = 5, color = 'black'),column=0, position = "float")
            elif node.support < 1 and node.support >= 0.95:
                    node.add_face(CircleFace(radius = 5, color = 'dimgray'),column=0, position = "float")
            elif node.support < 0.95 and node.support >= 0.9:
                    node.add_face(CircleFace(radius = 5, color = 'gray'),column=0, position = "float")
            elif node.support < 0.9 and node.support >= 0.8:
                    node.add_face(CircleFace(radius = 5, color = 'gainsboro'),column=0, position = "float")
            
    t.dist = 0
    t.support = 0
    t.show(tree_style = ts)
    t.render(path + tree_string_file.split('.')[0] + '_Figure_color.tif', w = 1000, tree_style = ts)

    #collapsed_nodes_file.close()
```

```
520
```

# Count hits

count blast hits and the number of TE identified by their features for table 1

In [17]:

```
import os
genome_codes = os.listdir('psiblast_ugene_RESULTS/')
temp = []
for code in genome_codes:
    if not '.' in code and not code in ['RET', 'BAS', 'PAT']:
        temp.append(code)
genome_codes = temp

def table_file_name(code):
    return 'psiblast_ugene_RESULTS/' + code + '/' + code + '_table.out'

TOTAL_hits = 0
TOTAL_defined = 0
for code in genome_codes:
    try:
        lines = open(table_file_name(code),'r').readlines()
        lines = lines[4:-8]
        total_hits = len(lines)
        defined_hits = 0
        options = ['dirs1-like','pat1-like','PAT','kangaroo','viper_or_Ngaro']
        for line in lines:
            if line.split()[1] in options:
                defined_hits += 1
        print code, total_hits, defined_hits
        TOTAL_hits = TOTAL_hits + total_hits
        TOTAL_defined = TOTAL_defined + defined_hits
    except:
        print code, '0', '0'
        
print '    ', TOTAL_hits, TOTAL_defined
```

```
Hcon 40 7
Acal 64 9
Acas 29 4
Agam 1 0
Alyr 0 0
Asuu 1 0
Ath 0 0
Avit 0 0
Bmal 0 0
briC 33 8
Bxyl 0 0
C11sp 0 0
C5sp 0 0
Cang 11 0
Cbre 0 0
Cele 1 0
Cjap 62 2
Crem 16 3
Dimm 1 0
Dpul 301 58
Dsim 0 0
Dviv 0 0
Ebre 459 0
Gpal 0 0
Haor 7 0
Hbac 2 0
Hduj 58 0
Lgi 333 17
Lloa 0 0
Lsig 0 0
Mflo 0 0
Mhap 0 0
Minc 0 0
Nve 495 42
Nvi 101 9
Ooch 0 0
Otip 12 0
Ovol 1 0
Ppac 57 7
Pred 5 0
Rcul 267 6
Srat 0 0
Tmur 0 0
Tspi 0 0
Vcar 182 35
Wban 0 0
     2539 207
```
