# Supplementary material for: The Evolution of Tyrosine-Recombinase Elements in Nematoda
Source: PLoS One. 2014 Sep 8;9(9):e106630. doi: 10.1371/journal.pone.0106630 (PMC4157794; doi:10.1371/journal.pone.0106630)
Supplement: Figure S1 — The phylogenetic classification of the recovered YREs. This phylogeny was reconstructed using only YR sequences from elements with defined borders (also available as Figure 2B), with a midpoint root (white background). Clades from the full YR tree (in grey) are presented next to reduced tree clades with which they share leaves. Large font black leaves are shared between the full and reduced YR trees. Large font green leaves are additional reference sequences. Small font leaves from the full tree (in grey) were added to the leaf count of the corresponding reduced tree clade. Only full tree clades with sh-like support >0.9 were considered. Full tree clades that included long branches were removed if they had sh-like support <0.95. The branch-length cutoff was four times the median branch-length of the clade. Leaf names include the species code (as in Tables 1 and S1), a unique number and the feature based classification. The unique number is the start position of the YR domain on its contig. table.out files in the pipeline results folder (http://dx.doi.org/10.6084/m9.figshare.1004150) provide access to the complete element information using the species code and the unique number. The unique number provides access to the element's diagram in the same folder. (PDF) [file pone.0106630.s001.pdf]

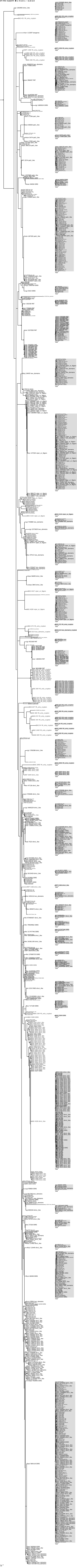
sh-like support: 1 0.9-1 0.8-0.9
